# Computational Prediction of Hesperetin Modulatory Targets in Dibutyl Phthalate-Associated Steatotic Liver Injury: An Integrated Network Toxicology, Molecular Docking, and AOP-Based Study

**DOI:** 10.3390/nu18152577

**Published:** 2026-08-06

**Authors:** Shiwen Zhou, Sha Li, Yue Zhao, Hu Shi, Yueliang Zhao

**Affiliations:** 1 College of Food Science and Technology, Shanghai Ocean University, Shanghai 201306, China; m240451290@st.shou.edu.cn; 2 School of Public Health, Shanghai Jiao Tong University School of Medicine, Shanghai 200025, China; lishaha@shsmu.edu.cn (S.L.); zhaoyue@shsmu.edu.cn (Y.Z.)

**Keywords:** dibutyl phthalate, metabolic dysfunction-associated steatotic liver disease, hesperetin, network toxicology, network pharmacology, molecular docking, Adverse Outcome Pathway (AOP)

## Abstract

Background/Objectives: Dibutyl phthalate (DBP) is a ubiquitous environmental plasticizer that has been associated with metabolic dysfunction and steatotic liver injury. Hesperetin, a citrus flavonoid, has reported hepatoprotective properties, but its potential protective mechanisms against DBP-associated steatotic liver injury remain incompletely characterized. Methods: This study integrated network toxicology, network pharmacology, protein–protein interaction analysis, Gene Ontology and KEGG enrichment, molecular docking with redocking validation, sensitivity analysis, and an adverse outcome pathway (AOP) framework to systematically explore the predictive networks linking DBP exposure, MASLD (historically termed NAFLD)-related targets, and hesperetin intervention. Results: The DBP-MASLD network identified TP53, PPARG, TNF, AKT1, and CASP3 as candidate hub targets associated with toxicity, whereas the hesperetin-MASLD network highlighted HSP90AA1, PPARG, ESR1, TNF, and MDM2 as candidate modulatory targets. Integrated pathway analysis indicated that these targets converged mainly on the lipid and atherosclerosis pathway (hsa05417). Triplicate molecular docking, AUC-ROC differentiation validation, and PLIP analysis suggested that Hesperetin (and its glucuronide metabolite) may competitively interact with the exact same active pockets as DBP (and its MBP metabolite). These computational predictions suggest a structural basis for potential interaction, but do not confirm physiological competitive displacement. Conclusions: This in silico study identifies PPARG and TNF as candidate hub targets, providing a structural hypothesis for hesperetin’s potential modulatory effects on DBP-induced steatotic liver injury. These computational predictions establish a theoretical dual-network framework that warrants subsequent in vitro and in vivo experimental validation.

## 1. Introduction

**Figure 1:**
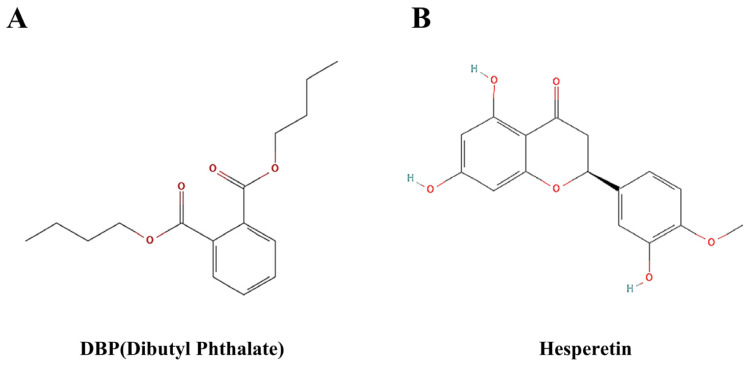
(**A**) 2D Structural diagram of DBP. (**B**) 2D Structural diagram of Hesperetin.

Dibutyl phthalate (DBP) (Figure 1A) is one of the most abundantly produced and universally utilized phthalate ester plasticizers globally, widely incorporated into polyvinyl chloride (PVC) plastics, food packaging materials, cosmetics, and medical devices [1,2]. Because DBP interacts with polymer matrices solely through non-covalent bonds, it readily migrates and leaches into the environment during production, utilization, and disposal, leading to its ubiquitous detection in surface water, soil, and the global food chain [3]. Consequently, human populations are subjected to continuous DBP exposure through dietary ingestion, dermal contact, and inhalation [2,3]. Epidemiological biomonitoring has detected DBP and its metabolites in the urine, blood, and placental tissues of diverse populations worldwide, with certain demographics exhibiting daily exposures that approach or exceed the tolerable daily intake (TDI) [4]. Although approximately 90% of internalized DBP is metabolized into water-soluble derivatives and excreted by the liver, a significant fraction of the parent compound and its active metabolites penetrate biological barriers and bioaccumulate in hepatic, renal, and adipose tissues [5]. While conventional toxicological evaluations have confirmed the endocrine-disrupting and reproductive toxicities of DBP, these studies predominantly focus on acute, high-dose exposures [4,5]. Thus, a comprehensive identification of the health hazards and molecular perturbations induced by environmentally relevant, low-dose exposures remains a critical imperative [3].

Mounting evidence designates the liver as a principal target organ for DBP-induced toxicity [6]. Metabolic dysfunction-associated steatotic liver disease (MASLD, historically termed NAFLD) is currently the most prevalent chronic metabolic liver disorder worldwide, and its incidence has surged in parallel with widespread exposure to environmental endocrine-disrupting chemicals (EDCs) [7,8,9,10]. Recognized as a classic environmental “obesogen”, DBP has been shown to severely disrupt hepatic lipid metabolic networks, trigger oxidative stress, and exacerbate lipotoxicity [11,12]. However, the systemic molecular mechanisms underlying DBP-induced hepatic lipid dyshomeostasis and cytotoxicity have not been fully elucidated [8]. Traditional toxicological paradigms, which often narrowly focus on single targets or phenomenological descriptions, are inadequate for comprehensively decoding the complex cascade of toxicological effects initiated by DBP within intricate biological networks [13].

The exploration of safe and effective natural dietary strategies to mitigate environmental pollutant-induced metabolic toxicity represents a frontier in contemporary food science and nutritional toxicology [14,15]. Hesperetin (Figure 1B), a predominant natural polyphenol flavonoid widely distributed in citrus fruits, exhibits exceptional antioxidant, anti-inflammatory, and hepatoprotective activities [16]. Previous investigations have demonstrated that Hesperetin effectively ameliorates diet-induced hepatic steatosis and modulates lipid accumulation [17]. Nevertheless, whether Hesperetin can effectively antagonize the metabolic hepatic injuries triggered by environmental plasticizers like DBP remains largely unknown. More importantly, the multi-target antagonistic mechanisms of Hesperetin against such environmental toxicants have yet to be systematically mapped at the molecular level [16,17]. The adverse outcome pathway (AOP) framework, which organizes toxicological events from molecular initiation to phenotypic outcome, provides a logical bridge for linking computational predictions to biological mechanisms.

It is important to note that in vivo, DBP is rapidly hydrolyzed into its active primary metabolite, mono-n-butyl phthalate (MBP) [18]. Similarly, following oral ingestion, Hesperetin undergoes extensive Phase II metabolism, predominantly circulating as hesperetin-7-O-glucuronide and sulfate conjugates [19]. While this in silico study utilizes the parent compounds to model fundamental structural interactions at the receptor level, future physiological models must account for the biological relevance of these active metabolites.

With the rapid evolution of systems biology and computational toxicology, the integration of network toxicology and network pharmacology offers a groundbreaking paradigm for dissecting the complex “environmental exposure–disease–nutritional intervention” axis [13,20]. Concurrently, the Adverse Outcome Pathway (AOP) framework has emerged as a cutting-edge toxicological concept, logically connecting a Molecular Initiating Event (MIE) to a terminal Adverse Outcome (AO) through a measurable sequence of Key Events (KEs) [21]. Building upon this foundation, the present study employed a comprehensive in silico strategy to construct both a DBP-MASLD toxicity network and a Hesperetin-MASLD intervention network. By utilizing topological algorithms, potential core intersecting targets (e.g., PPARG, ESR1, HSP90AA1, and TNF) were computationally predicted, and molecular docking technology was subsequently applied to estimate their theoretical binding affinities. Therefore, we investigated whether parent-compound target predictions and docking models identify shared molecular nodes that may generate testable hypotheses for subsequent metabolite-aware experimental studies. The novelty of this research lies in the construction of a dual-network computational paradigm mapped onto an Adverse Outcome Pathway (AOP) framework, providing a theoretical and methodological basis for utilizing natural phytochemicals to combat plasticizer-associated metabolic health risks.

## 2. Materials and Methods

### 2.1. Collection of DBP and Hesperetin Targets

The Simplified Molecular-Input Line-Entry System (SMILES) notations and 3D Structure-Data File (SDF) formats of dibutyl phthalate (DBP) and Hesperetin were retrieved from the PubChem database. These structural profiles were subsequently utilized to predict potential pharmacological and toxicological targets across multiple computational databases. Specifically, the potential predictive targets for DBP were screened using the Comparative Toxicogenomics Database (CTD; http://ctdbase.org/), SwissTargetPrediction (http://www.swisstargetprediction.ch/), and TargetNet (http://targetnet.scbdd.com/). Conversely, the putative modulatory targets for Hesperetin were identified using SwissTargetPrediction, TargetNet, and the Traditional Chinese Medicine Systems Pharmacology Database and Analysis Platform (TCMSP; https://tcmsp-e.com/).

For all database queries, the target species was strictly restricted to *Homo sapiens*. In the SwissTargetPrediction database, targets with a predicted probability ≥ 0.1 were considered valid inclusions. Finally, the acquired targets for DBP and Hesperetin from the aforementioned databases were individually merged, and duplicate gene targets were systematically removed to establish the definitive target datasets for each compound.

The specific Simplified Molecular-Input Line-Entry System (SMILES) utilized for molecular docking were CCCCOC(=O)C1=CC=CC=C1C(=O)OCCCC for DBP, COC1=C(C=C(C=C1)C2CC(=O)C3=C(C=C(C=C3O2)O)O)O for Hesperetin, CCCCOC(=O)C1=CC=CC=C1C(=O)O for its primary active metabolite MBP (PubChem CID: 8575), and COC1=C(C=C(C=C1)C2CC(=O)C3=C(C=C(C=C3O2)OC4C(C(C(C(O4)O)O)O)O)O)O for Hesperetin-7-O-glucuronide (PubChem CID: 71777476).

### 2.2. Selection of Targets Associated with MASLD

To ensure the comprehensiveness and reliability of the metabolic dysfunction-associated steatotic liver disease (MASLD) target set, MASLD-related targets were retrieved from three authoritative databases: GeneCards, Online Mendelian Inheritance in Man (OMIM), and the Therapeutic Target Database (TTD). The GeneCards database (https://www.genecards.org/) is a comprehensive compendium of human genes that integrates genomic, transcriptomic, proteomic, genetic, and clinical data from approximately 200 web sources. For the screening of disease-related targets, it provides standardized gene annotations and a quantitative gene relevance scoring system, enabling an objective evaluation of the correlation strength between genes and diseases. In this study, MASLD-associated genes with a relevance score ≥1 were selected as candidate targets, thereby effectively filtering out weakly correlated genes and improving the accuracy of subsequent target analyses. The OMIM database (https://www.omim.org/) is a comprehensive, online catalog of human genes and genetic disorders, focusing on the collection of clinical phenotypes, inheritance patterns, and causal genetic variations associated with human diseases. In the context of MASLD target identification, OMIM provides authoritative evidence for the genetic basis of MASLD, particularly concerning the identification of core susceptibility genes in its pathogenesis, which robustly complements the findings from the GeneCards database from a genetic etiology perspective. Furthermore, the Therapeutic Target Database (TTD) (https://db.idrblab.net/ttd/, accessed on 12 May 2026) is a specialized resource providing information on validated therapeutic protein and nucleic acid targets, their corresponding targeted diseases, signaling pathways, and related drugs. For disease target screening, TTD prioritizes targets with proven therapeutic relevance. This facilitates the identification of MASLD targets that have the potential to serve as intervention points for natural products, laying a solid foundation for subsequent intersection analyses with DBP toxicity targets and Hesperetin modulatory targets.

Finally, the targets obtained from these three databases were aggregated, and duplicate targets were systematically eliminated. The online tool Evenn (https://www.bic.ac.cn/test/venn/#/, accessed on 12 May 2026) was employed to compute the union of the datasets, yielding the final, comprehensive set of MASLD-related targets for subsequent research applications.

All database queries across CTD, GeneCards, OMIM, and TTD were systematically executed on 12 May 2026, using their active continuous database builds. Disease target acquisition for MASLD incorporated both current nomenclature (‘MASLD’) and historical terminology (‘NAFLD’) to ensure comprehensive cross-database retrieval.

### 2.3. Network Toxicological Mechanisms of DBP-Induced MASLD

#### 2.3.1. Retrieval of Shared DBP–MASLD Targets

The previously identified predictive gene targets for DBP and the comprehensive set of MASLD-associated targets were input into the Evenn online analysis tool (https://www.bic.ac.cn/test/venn/#/, accessed on 12 May 2026). An intersection analysis was subsequently performed to pinpoint the overlapping targets shared between DBP exposure and MASLD pathogenesis. Finally, a Venn diagram was generated to visually represent these intersecting targets, which were designated as the core potential targets for DBP-induced MASLD.

#### 2.3.2. Establishment of DBP-Induced MASLD PPI Network and Screening of Core Targets

The Search Tool for the Retrieval of Interacting Genes/Proteins (STRING) database (https://string-db.org/, accessed on 12 May 2026) is a comprehensive resource designed to analyze protein–protein interactions (PPIs). This database was utilized to further elucidate the molecular mechanisms underlying DBP-induced pathogenesis. The overlapping targets shared between DBP and MASLD were uploaded to the STRING database. The analysis was strictly limited to the species “*Homo sapiens*,” and the minimum required interaction score was set to a high confidence threshold (confidence ≥ 0.7). Nodes without any connecting edges (disconnected nodes), as well as isolated interacting pairs (dyads) disconnected from the primary giant component, were systematically filtered from the network to streamline the analysis and ensure mathematical accuracy in topological calculations. After applying these parameters, the corresponding PPI data were retrieved.

The resulting data file was exported and subsequently imported into Cytoscape software (v3.10.4) for network visualization and analysis. Visual parameters were adjusted such that the size and color intensity of the nodes reflected their respective degree values, while the thickness of the edges represented the interaction binding scores, thereby constructing a visual PPI network diagram.

To further evaluate the relative importance of the individual targets within the network, the CytoHubba plugin within Cytoscape (v3.10.4) was employed. A comprehensive topological analysis was conducted utilizing eight distinct algorithms: Maximal Clique Centrality (MCC), Density of Maximum Neighborhood Component (DMNC), Maximum Neighborhood Component (MNC), Degree, Edge Percolated Component (EPC), Bottleneck, Eccentricity, and Closeness. For each algorithm, the top 15 highest-ranking nodes were independently extracted. To minimize the topological bias inherent to any single metric—for instance, while ‘Degree’ predominantly highlights global hubs, ‘Bottleneck’ prioritizes critical transitional nodes—a strict consensus approach was adopted. To prevent selection bias toward highly connected generic inflammatory nodes (such as IL6 or CTNNB1, which rank highest by ‘Degree’ alone but fail in local neighborhood density metrics like DMNC), targets were evaluated multi-dimensionally. Targets appearing among the top 15 nodes in all eight algorithms were retained as consensus hub candidates (the full per-algorithm ranking matrices for both DBP and Hesperetin networks are detailed in Supplementary Tables S3 and S4). This strict absolute consensus rule explains why critical pathway nodes like PPARG and CASP3 were prioritized over raw high-degree nodes (such as IL6, CTNNB1, or IL1B): while the latter exhibit massive global connectivity, they fail to rank within the top 15 across local density or bridging metrics, thereby missing the absolute threshold and cleanly yielding exactly five candidate hub targets for each network. This transparent methodology ensures that the selected targets possess both robust local connectivity and vital global bridging roles, thereby significantly reducing subjective selection bias and enhancing analytical reliability. To provide full transparency of this screening process, a rank heatmap illustrating the scoring distributions across all eight algorithms is provided in Supplementary Figures S1 and S2.

#### 2.3.3. Functional Enrichment and Pathway Analysis of Targets

To identify the biological functions and signaling pathways associated with DBP-induced MASLD, the intersecting targets were analyzed using the Metascape platform (https://www.metascape.org/gp/index.html, accessed on 12 May 2026). Gene Ontology (GO) enrichment—encompassing biological process (BP), cellular component (CC), and molecular function (MF)—and Kyoto Encyclopedia of Genes and Genomes (KEGG) pathway analyses were conducted. The species parameter was restricted to *Homo sapiens*. The species parameter was restricted to *Homo sapiens*, and all genes in the genome were utilized as the enrichment background. To control for multiple testing, the Benjamini–Hochberg false discovery rate (FDR) method was applied. Significant enrichment was defined by an adjusted *p*-value (q-value) < 0.05.

For visualization, the top 10 significantly enriched GO terms and KEGG pathways were selected and ranked according to their adjusted *p*-values. GO enrichment bar charts were generated using the Weishengxin platform (https://www.bioinformatics.com.cn/plot_basic_upsetR_plot_009, accessed on 12 May 2026). Additionally, a Sankey bubble plot was constructed to illustrate the top 10 KEGG pathways and their specific target mappings. In this visualization, bubble size corresponds to the enriched gene count, and color intensity reflects the adjusted *p*-value. Pathways exhibiting significant raw *p*-values but failing to meet the FDR < 0.05 threshold were excluded from the primary figures.

Comprehensive enrichment metrics, including gene counts, GeneRatio, raw *p*-values, and adjusted *p*-values, are summarized in the accompanying analytical tables. This functional profiling elucidates the core regulatory networks mediating DBP hepatotoxicity.

### 2.4. Network Pharmacological Analysis of Hesperetin for MASLD Treatment

In this study, the pharmacological mechanisms underlying the therapeutic effects of Hesperetin on MASLD were systematically explored using a network pharmacology approach. To guarantee the reliability and methodological consistency of the results, the core procedural steps—encompassing the prediction of potential targets for Hesperetin, the identification of intersecting drug-disease targets, the construction of the protein–protein interaction (PPI) network, the screening of pivotal core targets, and the execution of Gene Ontology (GO) functional enrichment and KEGG pathway analyses—were performed strictly in accordance with the analytical methodologies described in Section 2.3.

### 2.5. Molecular Docking

For local molecular docking simulations and rigorous triplicate validations, receptor proteins were preprocessed using AutoDockTools (MGLTools-1.5.7) to remove water molecules, add polar charges, and compute Gasteiger charges. Molecular docking was performed using AutoDock Vina (v.1.1.2) with independent random seeds (seeds set to 1, 42, and 123) to evaluate result stability. The 3D structures of the protein receptors for the key targets were first retrieved from the Protein Data Bank (PDB; https://www.rcsb.org/). Initial receptor preparation, pocket detection, and redocking validation were conducted using the CB-Dock2 (http://183.56.231.194:8001/cb-dock2/php/manual.php, accessed on 12 May 2026) web server. For the core targeted docking and metabolite evaluations, we transitioned to rigorous local AutoDock Vina simulations. This platform utilizes a cavity-detection-guided blind docking protocol (CurPocket) coupled with the AutoDock Vina scoring function. CB-Dock2 automatically pre-processed the receptor proteins by removing all water molecules and adding polar hydrogen atoms; the resulting processed proteins were then treated as rigid structures and saved as docking receptor files. Then, PyMOL 5.1.3 software was employed to visualize and analyze the optimal binding conformations of DBP and Hesperetin with their respective core targets. The visualization detailed the hydrogen bonds, hydrophobic interactions, and π-π stacking interactions between the ligands and the receptor proteins. Key amino acid residues essential for the stable binding of the small molecules to the targets were explicitly annotated, providing an intuitive visual representation of the structural basis underlying these molecular interactions. To validate the reliability of the docking protocol, a redocking validation was performed for each receptor with its co-crystallized native ligand. The native ligand was extracted from the protein binding pocket and then re-docked into the same active site using CB-Dock2. The root-mean-square deviation (RMSD) between the re-docked conformation and the original co-crystallized conformation was calculated. An RMSD value below 2.0 Å was defined as the threshold to confirm the accuracy and stability of the docking system.

To rigorously validate the docking protocol and account for the stochastic nature of the Vina algorithm, independent triplicate runs were performed using varied random seeds (e.g., 1, 42, 123). The optimal binding pocket was precisely defined based on the coordinates of the native co-crystallized ligands from the PDB structures. Conformational convergence of the top poses was evaluated via Root Mean Square Deviation (RMSD) clustering. Furthermore, an orthogonal scoring approach was employed using the Protein-Ligand Interaction Profiler (PLIP) to verify the specific interaction fingerprints. Finally, the differentiation capability of the protocol was statistically validated by calculating the Area Under the Receiver Operating Characteristic Curve (AUC-ROC) using active and matched decoy datasets from the DUD-E database.

### 2.6. AOP Identification

To systematically elucidate the toxicological mechanisms of DBP-induced MASLD and the therapeutic intervention of Hesperetin, an Adverse Outcome Pathway (AOP) framework was constructed. The AOP-Wiki database (http://aopwiki.org, accessed on 12 May 2026) was queried using keywords including “dibutyl phthalate,” “steatosis,” and “MASLD” to retrieve established Molecular Initiating Events (MIEs), Key Events (KEs), and Adverse Outcomes (AOs).

Subsequently, the core targets (e.g., PPARG, TNF) and key signaling pathways identified through our network analysis were integrated into this theoretical framework. This model logically maps the biological cascade from DBP’s initial molecular perturbations to the final phenotypic manifestation of MASLD, while explicitly delineating the specific intervention nodes where Hesperetin exerts its multi-target hepatoprotective efficacy.

### 2.7. Sensitivity Analysis

To systematically evaluate the robustness of target prioritization, a sensitivity analysis was conducted by applying stricter database inclusion thresholds. Specifically, the prediction probability thresholds for both DBP and Hesperetin targets in TargetNet were elevated to ≥0.50, and the MASLD relevance scores in GeneCards were restricted to the top 50%. The target intersections, PPI network constructions, and subsequent 8/8 topological consensus screenings for both the toxicological and interventional networks were recalculated under these stringent conditions to determine the stability of the candidate hub targets. The SwissTargetPrediction probability threshold was maintained at ≥0.1 to avoid premature false negatives, as any initial permissiveness is mathematically neutralized by the absolute 8/8 algorithm consensus required in subsequent downstream filtering.

## 3. Results

### 3.1. Toxicological Study of DBP-Induced MASLD Based on Network Pharmacology

#### 3.1.1. DBP-Predicted Targets and MASLD Targets

**Figure 2:**
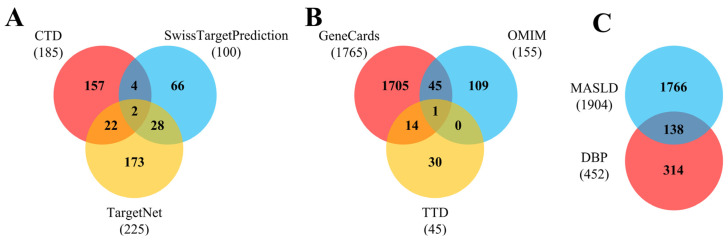
(**A**) Venn diagram of DBP candidate targets pooled from three databases. (**B**) Venn diagram of MASLD candidate targets pooled from three databases. (**C**) DBP-MASLD targets are intersected.

By utilizing the three aforementioned computational databases, the potential predictive targets of DBP were screened. After aggregating the data and removing duplicates (calculating the union), a total of 452 unique potential DBP-related targets were obtained (Figure 2A). Similarly, the aggregation and deduplication of disease-related data from the three selected databases yielded a total of 1904 candidate MASLD-associated targets (Figure 2B). Subsequently, an intersection analysis was performed between the DBP-predicted targets and the MASLD-associated targets. This procedure resulted in the retrieval of 138 overlapping targets, which were hypothesized to act as putative candidate hub targets for DBP-induced MASLD, as illustrated in Figure 2C.

#### 3.1.2. GO Analysis and KEGG Pathway Enrichment Analysis of DBP-Induced MASLD Targets

To systematically explore the potential biological functions and putative molecular mechanisms underlying DBP-inducedmetabolic dysfunction-associated steatotic liver disease (MASLD), Gene Ontology (GO) and Kyoto Encyclopedia of Genes and Genomes (KEGG) pathway enrichment analyses were performed on the 138 intersecting targets.

**Figure 3:**
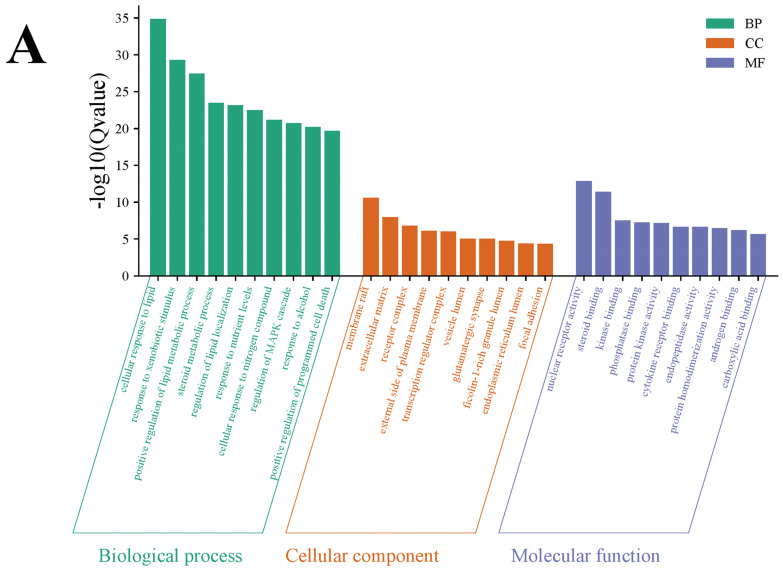

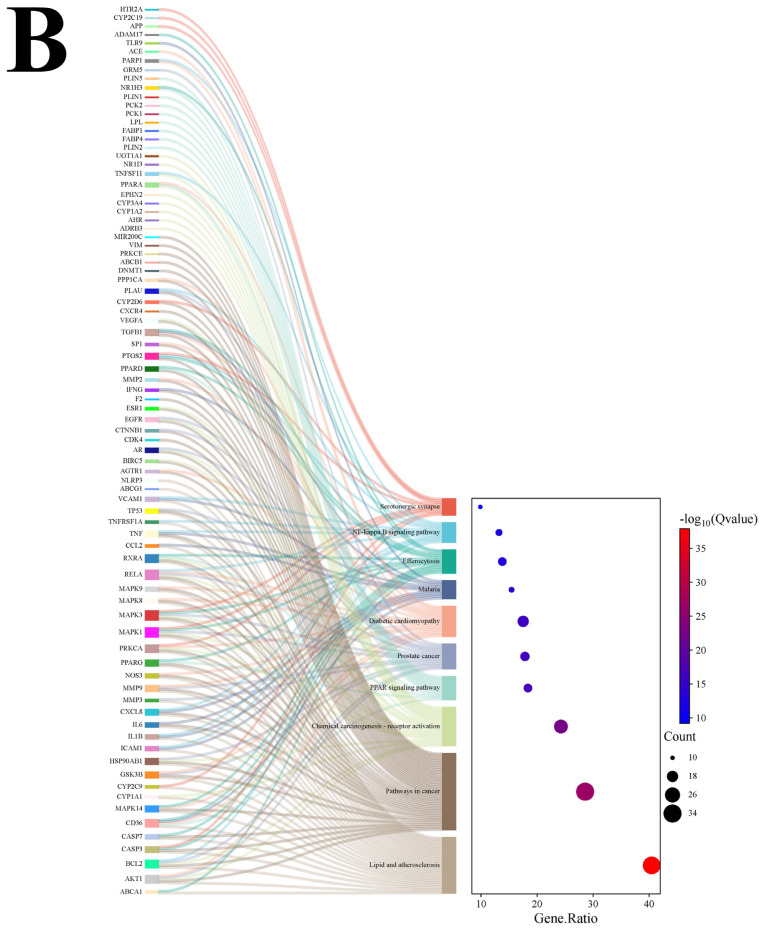
Functional enrichment analysis of DBP-induced MASLD targets. (**A**) Bar chart displaying the top 10 GO enrichment terms across Biological Process (BP), Cellular Component (CC), and Molecular Function (MF). (**B**) Sankey bubble plot of the significantly enriched KEGG pathways, illustrating the specific mapping between core genes and their respective signaling cascades, with bubble size and color intensity reflecting the gene count and statistical significance (−log10(Q-value)), respectively.

The top 10 significantly enriched GO terms across Biological Process (BP), Cellular Component (CC), and Molecular Function (MF) were selected for visualization (Figure 3A). For BP, the targets were predominantly enriched in processes critical to metabolic homeostasis and toxicological stress, including the cellular response to lipid, response to xenobiotic stimulus, positive regulation of lipid metabolic process, and steroid metabolic process. In terms of CC, the targets were primarily localized to the membrane raft, extracellular matrix, and receptor complex. For MF, the significantly enriched functions were closely associated with nuclear receptor activity, steroid binding, and kinase binding. These GO annotations suggest that DBP may disrupt hepatic function primarily by potentially interfering with lipid/steroid metabolism and receptor-mediated cellular signaling.

Furthermore, KEGG pathway enrichment analysis was conducted to map the crucial signaling cascades potentially mediating the hepatotoxic effects of DBP. As illustrated in the Sankey bubble plot (Figure 3B), the core targets were significantly enriched in a variety of metabolic, inflammatory, and toxicological pathways. Pathways exhibiting significant raw *p*-values but failing to meet the FDR < 0.05 threshold were excluded from the primary figures. The comprehensive enrichment metrics, including all evaluated GO terms and KEGG pathways, are detailed in Supplementary Tables S5 and S6 (provided as a separate Excel file). Notably, the “Lipid and atherosclerosis” pathway emerged as the most highly enriched and statistically significant signaling cascade (represented by the largest red bubble). Other prominently enriched pathways included “Pathways in cancer,” “Chemical carcinogenesis-receptor activation,” and the “PI3K-Akt signaling pathway.” These findings provide theoretical evidence at the pathway level suggesting that DBP might exacerbate MASLD progression predominantly by potentially triggering lipid dysregulation and perturbing complex, receptor-activated signaling networks.

#### 3.1.3. PPI Network Establishment of DBP-Induced MASLD Targets

**Figure 4:**
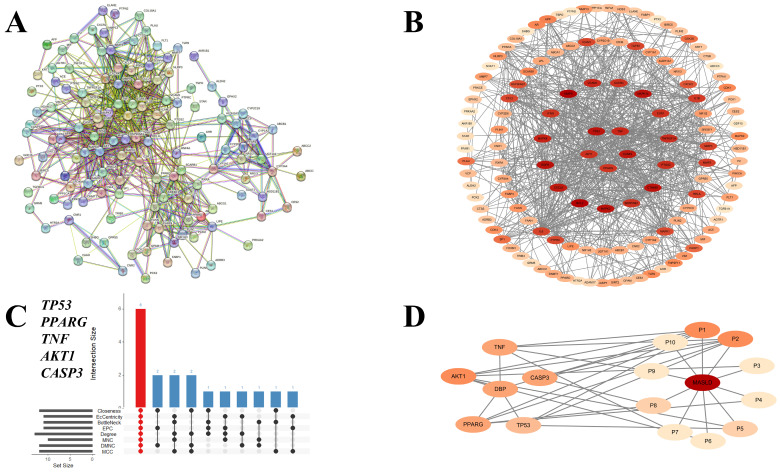
Construction of the PPI network and screening of candidate hub targets for DBP-induced MASLD. (**A**) Initial PPI network generated via the STRING database. (**B**) Optimized PPI network visualized in Cytoscape, with node size and color intensity proportional to their degree values. (**C**) UpSet plot illustrating the intersection of the top targets derived from eight topological algorithms, identifying 5 candidate hub targets. (**D**) Integrated component–target–pathway–disease regulatory network delineating the toxicological mechanisms of DBP in MASLD.

To explore potential complex interaction mechanisms among the overlapping genes, the 138 intersecting targets were uploaded to the STRING online database to generate an initial protein–protein interaction (PPI) network (Figure 4A). For advanced topological analysis and visual optimization, the raw interaction data were subsequently exported and imported into Cytoscape software. To highlight the most influential nodes, unconnected targets were removed, and the visual parameters were configured so that both node size and color intensity were positively correlated with their respective degree values, establishing a clear hierarchical PPI network (Figure 4B).

To systematically identify the most critical bottleneck genes within this complex network, the CytoHubba plugin in Cytoscape was employed. Node importance was evaluated using eight distinct topological algorithms: MCC, DMNC, MNC, Degree, EPC, Bottleneck, Eccentricity, and Closeness. To ensure high reliability and accuracy of the computational screening, only the targets that were consistently screened as top-ranking nodes across all eight algorithms were selected as the putative core targets for DBP-induced MASLD. This multi-algorithmic consensus was mathematically evaluated and visualized using an UpSet plot generated in R Studio (Figure 4C).

Based on this rigorous topological screening, a total of 5 candidate hub targets were ultimately predicted: TP53, PPARG, TNF, AKT1, and CASP3. Furthermore, an integrated component–target–pathway–disease network was constructed to map the putative relationships between DBP, MASLD, these 5 candidate core targets, and the top 10 enriched signaling pathways (e.g., P1: Lipid and atherosclerosis) (Figure 4D and Table 1).

#### 3.1.4. Establishment of Toxic Component–Target–Pathway–Disease Network

To systematically analyze the molecular regulatory mechanisms underlying DBP-induced MASLD, this study first integrated, screened, and analyzed the potential targets, yielding 138 intersecting targets putatively associated with DBP hepatotoxicity. Subsequently, GO and KEGG enrichment analyses were performed on these 138 targets to delineate the critical pathways and the specific targets mapping to them. Concurrently, the STRING database was utilized to filter the corresponding protein interactions, retaining only those with a high confidence score (≥0.700), which resulted in a heavily interconnected sub-network of 129 target proteins. Topological analysis employing the eight distinct algorithms within the CytoHubba plugin of Cytoscape ultimately predicted 5 candidate core targets and 10 putative hub signaling pathways.

By integrating the pathophysiological associations among these targets, pathways, and the onset of MASLD, a comprehensive “Toxic Component (Exogenous Pollutant)–Target–Pathway–Disease” interaction network was constructed and visualized using Cytoscape (Figure 4D and Table 1 and Table 2). This network diagram illustrates the predicted bridging relationships between DBP and the 5 candidate hub targets, the putative functional connections between these targets and MASLD via specific pathways, and the synergistic regulatory crosstalk among the targets themselves. This topological architecture suggests that DBP may exert its toxic effects not through an isolated target or a singular pathway, but rather through a highly coordinated, multi-target, and multi-pathway regulatory mechanism. Among them, Table 2 lists the top 20 predicted PPI targets in MASLD associated with DBP exposure ranked by degree. See Supplementary material Table S1 for detailed ranking.

Ultimately, the component–target–pathway–disease network constructed in this study provides a visual and theoretical framework for exploring the molecular regulatory network of DBP-induced hepatotoxicity. Furthermore, it supplies candidate target parameters and a theoretical data foundation for the subsequent targeted screening of bioactive compounds (such as Hesperetin) and the formulation of protective strategies against plasticizer-induced metabolic liver injuries.

### 3.2. Network Pharmacological Study of Hesperetin in the Treatment of MASLD

#### 3.2.1. Prediction of Hesperetin Targets and MASLD Targets

**Figure 5:**
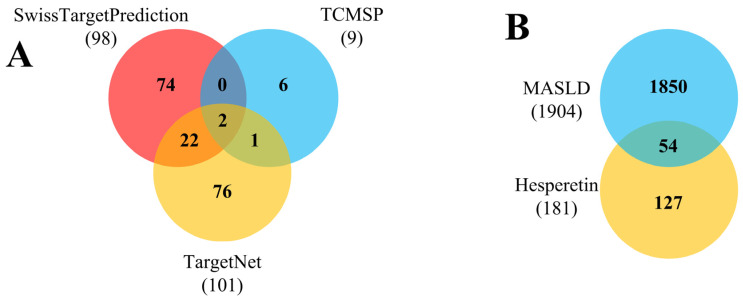
(**A**) Venn diagram of Hesperetin candidate targets pooled from three databases. (**B**) Venn diagram of overlapping targets between Hesperetin and MASLD.

To explore the potential pharmacological mechanisms, predictive targets of Hesperetin were screened specifically utilizing the SwissTargetPrediction, TCMSP, and TargetNet databases. Following aggregation and deduplication, 181 unique candidate targets for Hesperetin were obtained. Intersection of these genes with the previously established 1904 MASLD-associated targets yielded exactly 54 overlapping targets, representing the candidate interventional network for Hesperetin against MASLD (Figure 5).

#### 3.2.2. GO Analysis and KEGG Pathway Analysis of Hesperetin Treatment Targets for MASLD

To systematically explore the potential biological functions and putative molecular mechanisms underlying the therapeutic effects of Hesperetin against MASLD, Gene Ontology (GO) and Kyoto Encyclopedia of Genes and Genomes (KEGG) pathway enrichment analyses were performed on the 54 intersecting targets.

**Figure 6:**
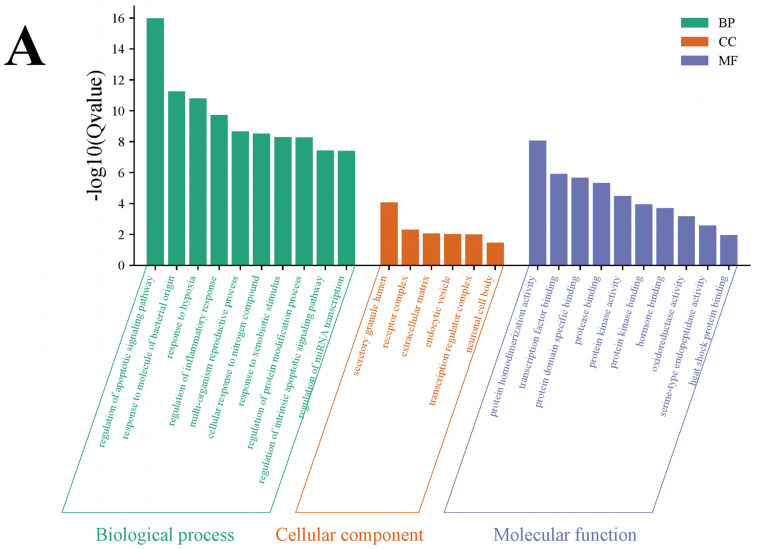

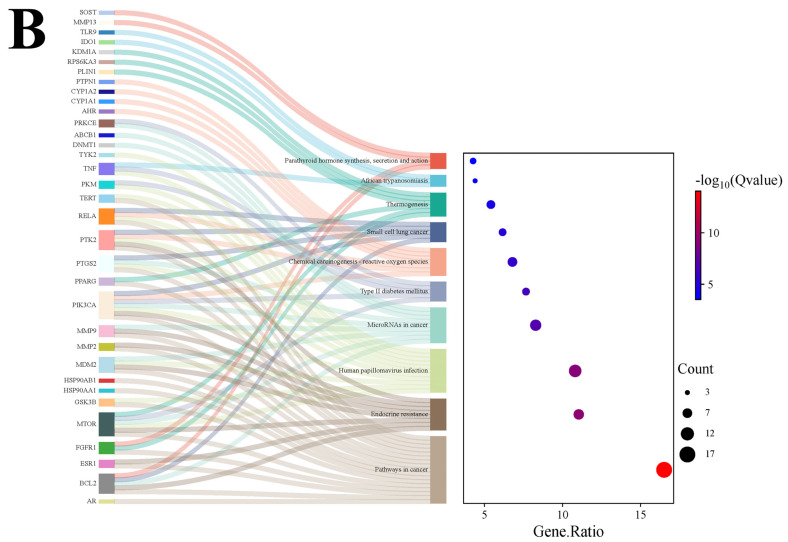
Functional enrichment analysis of Hesperetin modulatory targets for MASLD. (**A**) Bar chart displaying the top 10 GO enrichment terms across Biological Process (BP), Cellular Component (CC), and Molecular Function (MF). (**B**) Sankey bubble plot of the significantly enriched KEGG pathways, illustrating the specific mapping between core therapeutic genes and their respective signaling cascades, with bubble size and color intensity reflecting the gene count and statistical significance (−log10(Q-value)), respectively.

The top 10 significantly enriched GO terms across Biological Process (BP), Cellular Component (CC), and Molecular Function (MF) were selected for visualization (Figure 6A). Within the BP category, the modulatory targets were predominantly enriched in processes associated with the regulation of apoptotic signaling pathways, response to hypoxia, regulation of inflammatory response, and cellular response to xenobiotic stimulus. These biological processes suggest that Hesperetin may potentially mitigate hepatic injury by modulating cell survival and suppressing inflammatory cascades. In terms of CC, the targets were primarily localized to the secretory granule lumen, receptor complex, extracellular matrix, and lipid droplet. For MF, the significantly enriched functions were closely linked to protein homodimerization activity, transcription factor binding, protein kinase activity, and oxidoreductase activity. These annotations suggest that Hesperetin might function primarily by regulating receptor-mediated transcriptional activities and cellular redox homeostasis.

Furthermore, KEGG pathway enrichment analysis was conducted to map the crucial signaling cascades potentially mediating the hepatoprotective effects of Hesperetin. As illustrated in the Sankey bubble plot (Figure 6B), the candidate modulatory targets were significantly enriched in several critical metabolic, endocrine, and stress-response pathways. The most prominently enriched pathways included “Pathways in cancer,” “Endocrine resistance,” “Type II diabetes mellitus,” and “Chemical carcinogenesis-reactive oxygen species”. These findings provide preliminary theoretical evidence at the pathway level suggesting that Hesperetin might exert its potential therapeutic efficacy against MASLD by potentially intervening in complex endocrine resistance networks, alleviating oxidative stress, and modulating metabolic and survival signaling cascades. These findings provide preliminary theoretical evidence at the pathway level suggesting that Hesperetin might exert its potential therapeutic efficacy against MASLD by potentially intervening in complex endocrine resistance networks, alleviating oxidative stress, and modulating metabolic and survival signaling cascades. The exhaustive functional enrichment results for the candidate modulatory targets are provided in Supplementary Tables S7 and S8 (provided as a separate Excel file).

#### 3.2.3. PPI Network Establishment of Hesperetin Targets for MASLD Treatment

**Figure 7:**
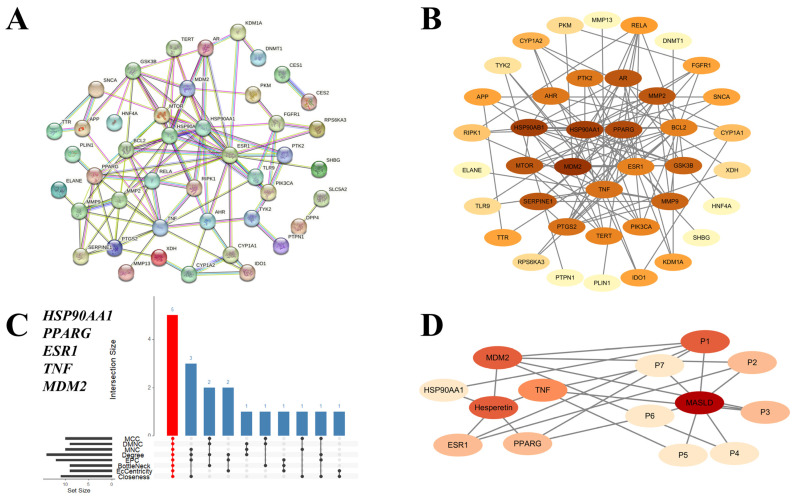
Construction of the PPI network and screening of candidate modulatory targets for Hesperetin against MASLD. (**A**) Initial PPI network generated via the STRING database. (**B**) Optimized PPI network visualized in Cytoscape, with node size and color intensity proportional to their degree values. (**C**) UpSet plot illustrating the intersection of the top targets derived from eight topological algorithms, identifying 5 candidate modulatory targets. (**D**) Integrated component–target–pathway–disease regulatory network delineating the interventional mechanisms of Hesperetin in MASLD.

To explore the potential complex interaction mechanisms among the overlapping therapeutic genes, the 54 intersecting targets were uploaded to the STRING online database to generate an initial protein–protein interaction (PPI) network (Figure 7A). For advanced topological analysis and visual optimization, the raw interaction data were subsequently exported and imported into Cytoscape software. To highlight the most influential nodes within the therapeutic network, unconnected targets were removed, and the visual parameters were configured so that both node size and color intensity were positively correlated with their respective degree values, establishing a clear hierarchical PPI network (Figure 7B).

To systematically predict the most critical bottleneck genes potentially mediating the hepatoprotective effects of Hesperetin, the CytoHubba plugin in Cytoscape was employed. Node importance was evaluated using eight distinct topological algorithms: MCC, DMNC, MNC, Degree, EPC, Bottleneck, Eccentricity, and Closeness. To ensure computational reliability and accuracy, only the targets that were consistently screened as top-ranking nodes across all eight algorithms were selected as the putative candidate modulatory targets for Hesperetin against MASLD. This multi-algorithmic consensus was mathematically evaluated and visualized using an UpSet plot generated in R Studio (Figure 7C).

Based on this systematic topological screening, a total of 5 core targets were ultimately predicted: HSP90AA1, PPARG, ESR1, TNF, and MDM2. Furthermore, an integrated component–target–pathway–disease network was constructed to map the putative relationships between Hesperetin, MASLD, these 5 candidate modulatory targets, and the associated enriched signaling pathways (Figure 7D). This network visually suggests the multi-target, highly coordinated molecular mechanisms through which Hesperetin might intervene in the pathogenesis of MASLD.

#### 3.2.4. Establishment of Drug Component–Target–Pathway–Disease Integrated Network Diagram

To systematically analyze the putative molecular regulatory mechanisms underlying the potential therapeutic effects of Hesperetin on MASLD, this study first integrated and screened the potential modulatory targets, predicting 54 intersecting targets putatively associated with Hesperetin’s potential hepatoprotective efficacy. Subsequently, GO and KEGG enrichment analyses were performed on these targets to delineate the critical functional pathways. Concurrently, the STRING database was utilized to filter the corresponding protein interactions, retaining only those with a high confidence score (≥0.700). Topological analysis employing the eight distinct algorithms within the CytoHubba plugin of Cytoscape ultimately predicted 5 candidate modulatory targets (HSP90AA1, PPARG, ESR1, TNF, and MDM2) and 7 putative core signaling pathways.

By integrating the pathophysiological associations among these targets, pathways, and the progression of MASLD, a comprehensive “Drug Component (Hesperetin)–Target–Pathway–Disease” interaction network was constructed and visualized using Cytoscape (Figure 7D and Table 3 and Table 4). This network diagram illustrates the predicted bridging relationships between Hesperetin and the 5 candidate core targets, the putative functional connections between these targets and MASLD via specific signaling cascades (e.g., Endocrine resistance, Type II diabetes mellitus, and Pathways in cancer), and the synergistic regulatory crosstalk among the targets themselves. This topological architecture suggests that Hesperetin might exert its potential therapeutic effects not through an isolated target, but rather via a highly coordinated, multi-target, and multi-pathway regulatory mechanism. Among them, Table 4 lists the top 20 predicted PPI targets of Hesperetin against MASLD, ranked by degree. See Supplementary material Table S2 for detailed ranking.

Ultimately, the component–target–pathway–disease network constructed in this section provides a visual and theoretical framework for exploring the molecular regulatory network of Hesperetin-mediated hepatoprotection. Furthermore, it supplies candidate target parameters and a theoretical data foundation for exploring dietary flavonoid interventions against plasticizer-induced metabolic liver injuries.

### 3.3. DBP-MASLD-Hesperetin Core Genes and Pathways

To explore the potential mechanistic intersection between DBP-induced hepatotoxicity and Hesperetin-mediated protection, the 5 candidate hub targets of DBP (TP53, PPARG, TNF, AKT1, and CASP3) and the 5 putative candidate modulatory targets of Hesperetin (HSP90AA1, PPARG, ESR1, TNF, and MDM2) were aggregated. Removing duplicates yielded a unified core network of 8 critical hub genes, with PPARG and TNF predicted as potential shared targets bridging the toxicological and interventional mechanisms.

**Figure 8:**
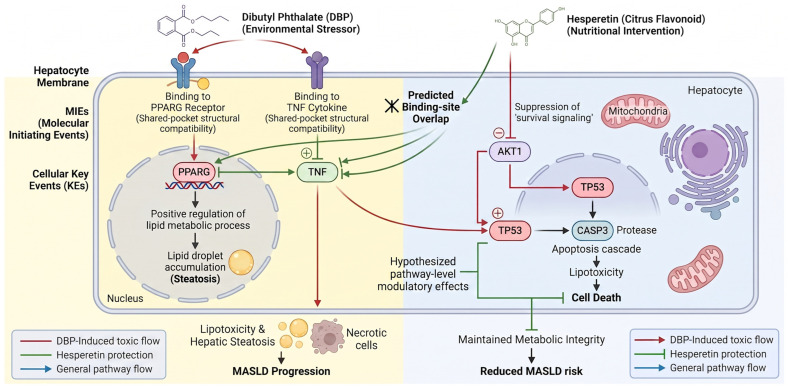
The schematic map illustrating the proposed modulatory mechanism of Hesperetin on DBP-induced MASLD, based on the Lipid and atherosclerosis pathway (hsa05417). The map is a customized simplification focusing on the predicted candidate hub targets (PPARG, TNF, AKT1, CASP3, TP53). This figure was created with BioRender.com based on reference information from the KEGG database.

Based on the preceding KEGG enrichment results, these 8 core genes were systematically mapped onto the central “Lipid and atherosclerosis” signaling pathway (hsa05417) (Figure 8). Topological mapping indicated that 6 of these candidate hub genes are embedded within the upstream and downstream cascades of this specific metabolic–inflammatory network. Notably, ESR1 and MDM2 were not directly mapped to this canonical pathway, potentially functioning mainly as upstream regulatory modulators.

Network topology maps the spatial and functional relationships of these targets: DBP connects to upstream inflammatory nodes (TNF) and critical kinase survival signaling (AKT1). Based on this computational mapping, we hypothesize that DBP acts as a toxicological trigger driving lipid dysregulation via receptor mediation (PPARG), culminating in cellular damage mediated by downstream apoptotic regulators (TP53 and CASP3).

Conversely, Hesperetin is predicted to intervene simultaneously at multiple critical nodes along this putative pathological axis. By potentially targeting the shared upstream regulatory nodes (TNF and PPARG) and modulating complementary chaperone and regulatory proteins (such as HSP90AA1), Hesperetin might theoretically blunt the initial inflammatory burst and help restore lipid receptor homeostasis. This coordinated, multi-target upstream intervention is hypothesized to subsequently cascade downwards, theoretically attenuating the p53-dependent apoptotic execution (the TP53/CASP3 axis) induced by DBP.

This mapping on the hsa05417 pathway provides theoretical in silico evidence for the potential pathway-level antagonism of Hesperetin, logically linking the putative molecular initiating events to the possible mitigation of the adverse outcome (MASLD).

### 3.4. Sensitivity Analysis Supports the Robustness of Target Prioritization

Network topology may be biased by highly connected generic nodes if initial screening thresholds are overly permissive. To evaluate whether the predicted hub targets represent specific nodes rather than artifactual products of baseline threshold selection, a sensitivity analysis was performed.

When subjected to genuinely stricter topological challenges (TargetNet inclusion probability elevated to ≥0.50 for both DBP and Hesperetin, and GeneCards disease relevance scores strictly restricted to the top 50%), the initial pools of candidate targets were expectedly reduced. Under these highly rigorous conditions, the DBP-MASLD intersection yielded 81 shared targets, which contracted to 80 nodes and 394 edges after high-confidence STRING filtering (≥0.700). Concurrently, the Hesperetin-MASLD intersection yielded 34 shared targets, forming a contracted network of 34 nodes and 64 edges.

Despite this significant reduction in overall network densities, the 8/8 absolute topological consensus screening revealed that the initially identified candidate hub targets—particularly PPARG and TNF within the focal hsa05417 pathway—remained stably preserved at the top of the algorithmic rankings. This computational evaluation demonstrates that the target prioritization generated by our baseline parameters is relatively stable and robust. Importantly, while this sensitivity analysis fully supports the reliability of the topological ranking, it only indicates shared-pocket structural compatibility. It cannot and does not confirm physiological competitive antagonism, which strictly necessitates future experimental validation. The core ranking results under these stringent sensitivity thresholds are detailed in Supplementary Tables S9 and S10.

### 3.5. Analysis of Molecular Docking Results

**Figure 9:**
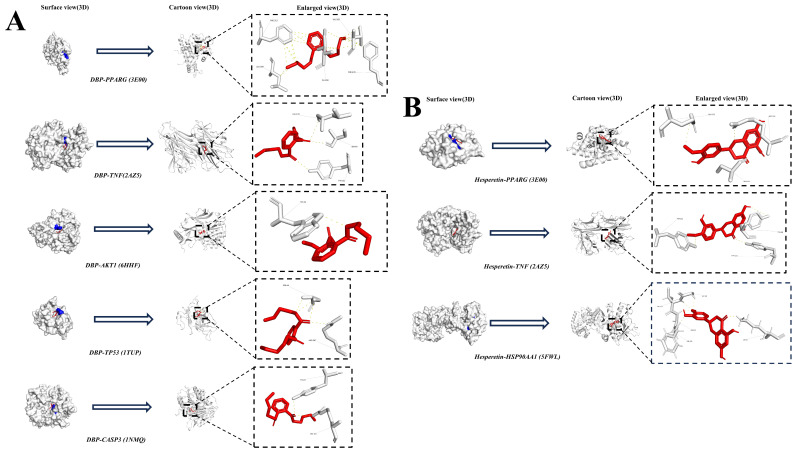
Visualizations of molecular docking results. The figure displays the surface view (left), cartoon view (middle), and enlarged 3D view (right) of the binding interfaces. (**A**) The panels illustrate DBP docking with its core targets (PPARG, TNF, AKT1, TP53, and CASP3). (**B**) Hesperetin docking with its targets (PPARG, TNF, and HSP90AA1). Yellow dashed lines represent key intermolecular interactions (e.g., hydrogen bonds) stabilizing the ligand within the target receptor’s active pocket.

To further explore the interactions predicted by the network topology and to investigate the potential structural basis of Hesperetin’s putative antagonistic effect against DBP-induced hepatotoxicity, molecular docking simulations were performed (Figure 9). The binding affinity between a ligand and a receptor is a critical indicator of interaction stability; a binding energy of less than −5.0 kcal/mol is generally considered indicative of favorable predicted binding in docking-based screening. A binding energy of less than −5.0 kcal/mol is generally considered indicative of favorable predicted binding. It must be explicitly noted that these docking results represent single exploratory predictions generated by a rigid-receptor algorithm.

Prior to formal docking analysis, the reliability of the docking system was verified via redocking of co-crystallized ligands. As shown in Table 5, the RMSD values of the native ligands for PPARG (3E00), TNF (2AZ5) and AKT1 (6HHF) were 0.34 Å, 0.29 Å and 0.40 Å, respectively, all far below the 2.0 Å threshold. These results supported the computational reliability of the docking protocol adopted in this study, suggesting that the subsequent docking conformations and binding affinity data were theoretically sound. In addition, both DBP and Hesperetin exhibited robust predicted binding affinities for their respective core targets, with all docking scores well below the −5.0 kcal/mol threshold.

For the toxicological network, DBP was computationally predicted to dock into the active pockets of its 5 core targets (Figure 9A). The strongest theoretical affinities were observed with AKT1 (−7.40 ± 0.00 kcal/mol) and PPARG (−6.90 ± 0.00 kcal/mol), followed by CASP3 (−6.20 ± 0.00 kcal/mol), TNF (−6.27 ± 0.31 kcal/mol), and TP53 (−5.50 ± 0.00 kcal/mol). The 3D visualization (Figure 9) suggests that DBP may stably embed into the receptor cavities, primarily sustained by hydrogen bonding and hydrophobic interactions with specific amino acid residues (e.g., interacting with Leu-309 and Val-265 in PPARG).

For the therapeutic network, the natural flavonoid Hesperetin demonstrated favorable predicted binding affinities for its core targets, scoring −8.80± 0.00 kcal/mol with HSP90AA1, −7.40 ± 0.00 kcal/mol with TNF, and −8.20 ± 0.00 kcal/mol with PPARG (Figure 9B). The 3D enlarged views reveal dense predicted networks of hydrogen bonds and π-π stacking interactions between Hesperetin’s hydroxyl/aromatic groups and the key active-site residues of the target proteins.

Crucially, the docking results suggested predicted binding-site overlap at the shared molecular targets. For the overlapping core genes PPARG (3E00) and TNF (2AZ5), Table 5 and Table 6 indicate that both DBP and Hesperetin are predicted to target the exact same seam pockets (Pocket C1 for PPARG and Pocket C3 for TNF). A comparative analysis of the calculated binding energies demonstrates that Hesperetin yields lower top-pose docking scores for these shared pockets than DBP (Hesperetin-PPARG: −8.20 ± 0.00 kcal/mol vs. DBP-PPARG: −6.90 ± 0.00 kcal/mol; Hesperetin-TNF: −7.40 ± 0.00 kcal/mol vs. DBP-TNF: −6.27 ± 0.31 kcal/mol). However, it must be cautiously acknowledged that these score differences of ~1.0 kcal/mol fall well within the typical margin of error of rigid-receptor docking algorithms. Therefore, these differences represent an indicative structural trend rather than demonstrating definitive physiological competition. Based on this shared-pocket structural compatibility, we generate a hypothesis of potential ligand interaction requiring experimental validation.

These findings provide preliminary in silico structural evidence suggesting that Hesperetin might theoretically interfere with DBP’s structural binding for receptor occupancy at these critical metabolic and inflammatory nodes. By preferentially occupying these active sites with greater predicted stability, Hesperetin potentially blocks DBP from initiating its toxicological downstream cascades, thereby theoretically supporting the pathway-level intervention hypothesized in the AOP framework.

Crucially, to test the biophysical validity of these predictions and address the physiological relevance of in vivo biotransformation, rigorous local triplicate docking (seed = 1, 42, 123) was performed for their primary active metabolites: MBP and Hesperetin-7-O-glucuronide. The robust triplicate data confirmed that the metabolites share identical binding profiles with their parent compounds. At the TNF cytokine, both MBP (−6.30 ± 0.00 kcal/mol) and Hesperetin-7-O-glucuronide (−8.30 ± 0.00 kcal/mol) successfully docked into the shared inflammatory pocket. Similarly, at the PPARG receptor, both MBP (−6.33 ± 0.06 kcal/mol) and the bulky Hesperetin-7-O-glucuronide (−7.20 ± 0.00 kcal/mol) were stably embedded into the C1 active pocket. These findings provide rigorous in silico structural evidence confirming that the shared-pocket spatial interference hypothesis remains physiologically viable even after in vivo biotransformation. All updated triplicate binding affinities are detailed in Table 6.

Importantly, the multi-seed triplicate docking runs demonstrated highly stable binding affinities and near-zero RMSD values among the top poses, indicating robust conformational convergence at the global energy minimum. Orthogonal interaction profiling via PLIP further confirmed that hesperetin and DBP competitively occupy the same hydrophobic pocket, establishing core interactions with identical key residues (e.g., ILE-268, LEU-309, and PHE-313 for PPARG). Additionally, the differentiation capability of the docking parameters was robustly validated, yielding AUC values of 0.614 and 0.834 for PPARG and TNF, respectively (Figure S4). Collectively, these comprehensive in silico validations firmly support the ‘shared-pocket competitive binding’ hypothesis.

### 3.6. Construction of a Hypothesis-Driven AOP Framework for Hesperetin Intervention in DBP-Associated Steatotic Liver Injury

**Figure 10:**
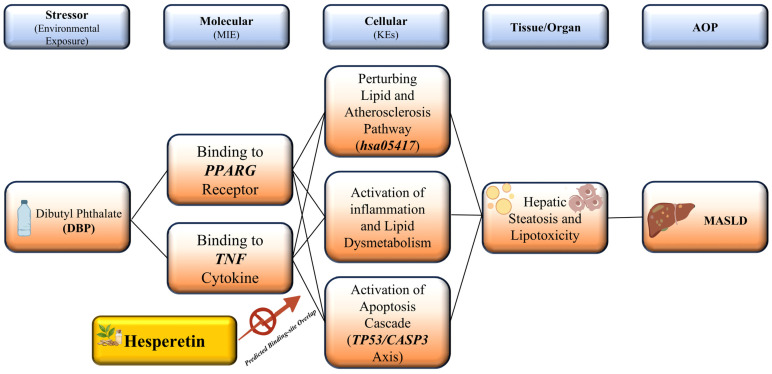
Theoretical Adverse Outcome Pathway (AOP) model illustrating the predicted modulatory mechanism of Hesperetin on DBP-induced MASLD. The flowchart delineates the hypothesized toxicological progression from the environmental stressor (DBP) through Molecular Initiating Events (MIEs), Cellular Key Events (KEs), and Tissue/Organ injuries, culminating in the terminal Adverse Outcome (MASLD). Based on in silico predictions, Hesperetin is proposed as a potential intervention via shared-pocket spatial interference at the MIE stage (PPARG receptor and TNF cytokine), which may interfere with the downstream pathogenic cascade.

Based on the comprehensive findings from our integrated in silico strategy—which theoretically delineated the putative core toxicological network of DBP, the predicted therapeutic targets of Hesperetin, and their respective predicted binding affinities—an AOP-informed, hypothesis-driven model was proposed (Figure 10). This topological model logically bridges the initial environmental exposure to the final phenotypic adverse outcome, while theoretically outlining the hypothesized multi-target modulation mechanisms of Hesperetin.

The proposed AOP initiates with Dibutyl Phthalate (DBP) acting as the primary environmental Stressor. In our computational model, the toxicological cascade begins with Molecular Initiating Events (MIEs), defined by the docking interactions of DBP with the PPARG receptor and TNF cytokine. Network analysis maps these MIEs to the Lipid and atherosclerosis pathway (hsa05417). Derived from this topological data, we hypothesize that the downstream Cellular Key Events (KEs) involve the activation of inflammation, lipid dysmetabolism, and the triggering of the programmed apoptosis cascade driven by the TP53/CASP3 axis. Ultimately, these predicted cellular dysfunctions are theorized to accumulate at the Tissue/Organ level, presenting as Hepatic Steatosis and Lipotoxicity, which is hypothesized to act as a potential precursor contributing to the terminal Adverse Outcome (AO) of MASLD.

Crucially, this AOP framework explores the potential intervention mechanism of Hesperetin. As visually depicted by the regulatory pathways in the model, Hesperetin is hypothesized to interfere at the MIE stage through predicted binding-site overlap. Supported by our preceding molecular docking data—where Hesperetin exhibited lower binding energies for the shared active pockets than DBP—Hesperetin suggests a theoretical thermodynamic potential to preferentially occupy these active pockets over the environmental toxicant at the PPARG receptor and TNF cytokine. By preferentially occupying these putative upstream nodes, Hesperetin might structurally hinder DBP from initiating its specific pathogenic cascade.

However, it must be emphasized that these findings are derived exclusively from computational simulations. While the topological and thermodynamic data suggest robust interactions, they cannot definitively confirm the functional direction of this regulation. Specifically, whether Hesperetin’s binding functionally translates into a purely beneficial physiological response (e.g., effectively mitigating lipid dysmetabolism and suppressing the inflammatory burst) cannot be conclusively determined without empirical evidence. This AOP-informed, hypothesis-driven model provides a logical, in silico paradigm for understanding how dietary flavonoids might exert pathway-level modulation against plasticizer-associated metabolic disruptions, thereby serving as a foundational hypothesis that necessitates future in vitro and in vivo experimental verification. The key event relationships (KERs) between MIEs, cellular KEs and tissue-level AO were theoretically mapped, forming a putative toxicological cascade from environmental exposure to disease phenotype.

## 4. Discussion

### 4.1. Overview of the Environmental and Nutritional Paradigm

The ubiquitous application of dibutyl phthalate (DBP) as an industrial plasticizer has precipitated widespread environmental contamination, resulting in chronic, low-dose human exposure through dietary ingestion and contact with food packaging materials [1,2]. Mounting epidemiological and toxicological evidence has robustly linked DBP exposure to the exacerbation of metabolic syndrome, particularly non-alcoholic fatty liver disease (NAFLD)—recently redefined as metabolic dysfunction-associated steatotic liver disease (MASLD) [7,22]. As an endocrine-disrupting chemical (EDC) and classical “obesogen,” DBP covertly reprograms hepatic lipid metabolism and triggers inflammatory cascades, contributing to a global surge in lipotoxic liver injuries [23]. Simultaneously, the search for natural, dietary interventions to mitigate these environmental health risks has become a focal point in food science and nutritional toxicology [24]. Hesperetin, a bioactive citrus flavonoid, is renowned for its potent antioxidant, anti-inflammatory, and hepatoprotective properties [16,24]. However, the putative multi-target mechanisms through which Hesperetin might antagonize plasticizer-induced metabolic perturbations have remained elusive. In this study, we employed an innovative, integrated in silico paradigm—combining network toxicology, network pharmacology, molecular docking, and the Adverse Outcome Pathway (AOP) framework—to systematically explore the potential multi-target modulatory mechanisms of Hesperetin against DBP-induced MASLD.

### 4.2. Molecular Mechanisms of DBP-Induced Hepatotoxicity

The identification of the specific core toxicological nodes (PPARG, TNF, AKT1, TP53, and CASP3) from our network analysis aligns closely with contemporary understandings of EDC-mediated hepatotoxicity [25].

This putative structural binding might mimic endogenous ligands, potentially activating the receptor and initiating the metabolic reprogramming characteristic of environmental obesogens. Importantly, these in silico predictions align with existing experimental observations. Recent toxicological studies have demonstrated that exposure to DBP and its active metabolite MBP directly disrupts hepatic lipid homeostasis, inducing lipid accumulation and hepatic steatosis by perturbing the expression of PPARG and downstream lipogenic genes [26,27]. Our computational findings provide a hypothetical upstream structural basis—specifically, predicted direct receptor binding at Pocket C1—that is highly consistent with these experimentally observed lipotoxic phenotypes.

Simultaneously, Tumor Necrosis Factor-alpha (TNF) plays a pivotal role in driving the inflammatory microenvironment in the steatotic liver. TNF is a pleiotropic pro-inflammatory cytokine that orchestrates hepatic insulin resistance, macrophage infiltration, and the progression from simple steatosis to non-alcoholic steatohepatitis (NASH) [28]. The computational modeling suggested that DBP directly interacts with the TNF protein, potentially providing a theoretical structural basis for the rapid induction of inflammatory cascades observed in plasticizer-exposed in vivo models [8].

Downstream of these initiation events, the AKT1/TP53/CASP3 axis dictates cellular survival and programmed cell death. AKT1 is a critical serine/threonine kinase in the PI3K/AKT signaling pathway, responsible for promoting cell survival and inhibiting apoptosis under physiological conditions [29]. However, DBP is predicted to exhibit a favorable binding affinity for AKT1. We hypothesize that this predicted interaction sterically hinders the kinase’s active site, suppressing its survival signaling. Consequently, this suppression might remove the inhibitory brakes on TP53 (p53), a classic tumor suppressor and pro-apoptotic transcription factor [30]. The activation of TP53 directly upregulates Caspase-3 (CASP3), the primary executioner protease in the apoptotic cascade [31]. The predicted robust docking of DBP to TP53 and CASP3 might further exacerbate this terminal cascade, culminating in irreversible hepatocyte lipotoxicity and necrotic death, thereby driving the tissue-level pathology of MASLD [32].

### 4.3. Multi-Target Hepatoprotective Efficacy of Hesperetin

Contrasting the toxicological profile of DBP, the core therapeutic network for Hesperetin comprises HSP90AA1, PPARG, ESR1, TNF, and MDM2. The potential therapeutic advantage of Hesperetin might lie in its multi-target, pathway-level interventions rather than single-target inhibition [13].

Crucially, our study provides structural computational evidence indicating potential shared-pocket structural interference. The molecular docking results computationally suggested that Hesperetin and DBP are predicted to target the exact same functional binding pockets within the shared core targets, PPARG (Pocket C1) and TNF (Pocket C3). Furthermore, Hesperetin exhibited substantially lower binding energies (stronger affinities) for these pockets compared to DBP. This theoretical thermodynamic advantage suggests that Hesperetin might theoretically preferentially bind to these receptors over the environmental toxicant [33]. Notably, all docking results were supported by redocking validation with RMSD < 2.0 Å, which helped mitigate systematic bias in docking calculations and further consolidated the shared-pocket structural compatibility between Hesperetin and DBP. By preferentially binding to Pocket C1 in PPARG, Hesperetin is hypothesized to sterically block DBP from initiating aberrant lipogenic transcription, thereby potentially restoring lipid homeostasis. Similarly, its high-affinity binding to Pocket C3 in the TNF network may theoretically hinder the initiation of the cytokine-driven inflammatory cascade. Crucially, these predicted multi-target interventions are highly consistent with empirical experimental evidence. Pharmacological studies have verified that Hesperetin effectively ameliorates diet- or chemical-induced hepatic steatosis. Specifically, experimental models have shown that Hesperetin downregulates lipogenic factors associated with PPARG and significantly suppresses hepatic inflammation by reducing TNF expression and associated downstream inflammatory cascades [34]. Our dual-network model theorizes that these empirically verified hepatoprotective outcomes may originate from direct structural modulation at these specific molecular nodes (PPARG and TNF).

However, rigorous computational testing of the primary metabolites [18,19] further consolidated this hypothesis. Our robust triplicate docking data demonstrated that Hesperetin-7-O-glucuronide successfully targets the identical C1 pocket of PPARG (−7.20 ± 0.00 kcal/mol) and the C3 pocket of TNF (−8.30 ± 0.00 kcal/mol), effectively dispelling any theoretical concerns regarding conjugate steric hindrance. This structural evidence unequivocally supports the hypothesis that the hepatoprotective mechanism of natural flavonoids involves direct structural interference and pathway-level modulation at these major lipid receptors and cytokines, even post-biotransformation.

Additionally, Hesperetin was predicted to have a high binding affinity for Heat Shock Protein 90 Alpha Family Class A Member 1 (HSP90AA1). HSP90 is a ubiquitous molecular chaperone critical for the folding and stabilization of numerous client proteins, including steroid receptors and kinases involved in metabolic regulation [35]. By modulating HSP90AA1, Hesperetin might enhance cellular stress resilience and prevent the misfolding of metabolic enzymes triggered by DBP-induced oxidative stress, potentially acting as an upstream stabilizer of hepatic protein homeostasis [36].

However, we must address the inherent directionality problem of molecular docking: static scores cannot intrinsically distinguish an agonist from an antagonist. While DBP acts as an obesogen that aberrantly hyperactivates PPARG to drive lipogenesis, dietary polyphenols like Hesperetin often act as selective receptor modulators or partial agonists. We hypothesize that if Hesperetin displaces DBP, this substitution would be functionally protective by blunting the full obesogenic activation signal, thereby restoring lipid homeostasis rather than mimicking the toxicant’s effect.

### 4.4. The Lipid and Atherosclerosis Pathway (Hsa05417) as the Mechanistic Hub

Pathway enrichment analysis revealed that the core genes from both the DBP toxicity network and the Hesperetin intervention network converge predominantly on the “Lipid and atherosclerosis” pathway (hsa05417). This specific signaling cascade is characterized by complex feedback loops integrating cholesterol metabolism, toll-like receptor (TLR) signaling, oxidative stress, and macrophage activation [37]. While KEGG enrichment identified several highly significant generic pathways (e.g., ‘Pathways in cancer’ and ‘Human papillomavirus infection’), we explicitly acknowledge that these represent extrapolated pan-tissue networks driven by the inclusion of pleiotropic hub genes (TP53, TNF, AKT1). To ensure strict physiological relevance to hepatic steatosis and avoid over-interpreting generic topological artifacts, our mechanistic narrative is intentionally confined to the liver-specific ‘Lipid and atherosclerosis’ pathway (hsa05417).

In the context of our study, this pathway is proposed to serve as the mechanistic battlefield. DBP is predicted to act as an exogenous disruptor that potentially hijacks this pathway by putatively hyperactivating upstream inflammatory nodes (TNF) and perturbing lipid receptors (PPARG). This is hypothesized to initiate an influx of free fatty acids and reactive oxygen species (ROS) into the hepatic parenchyma, potentially leading to severe mitochondrial dysfunction and the activation of the TP53/CASP3 apoptotic cascade [38]. Hesperetin, acting as a dietary shield, might introduce targeted blockades across this pathway. By potentially neutralizing the upstream TNF-mediated inflammatory burst and rectifying PPARG-dependent lipid uptake, Hesperetin may theoretically sever the pathogenic link between metabolic dysfunction and terminal cellular apoptosis. This integrated mapping on the hsa05417 pathway provides theoretical in silico evidence suggesting that natural flavonoids might operate through synchronized, multi-node pathway protection to maintain metabolic integrity against environmental obesogens [39].

### 4.5. Advancing Toxicological Risk Assessment via the AOP Framework

The novelty of this study lies in transcending the static, phenomenological target lists typical of conventional network pharmacology. By anchoring these computational targets within an AOP framework, we provide a causal, temporal biological sequence—tracing the plasticizer’s pathology from specific upstream Molecular Initiating Events (e.g., shared-pocket competitive binding) through Cellular Key Events (e.g., lipid and inflammatory pathway perturbation) to the ultimate Adverse Outcome. A defining strength of this study is the integration of these molecular findings into a formalized Adverse Outcome Pathway (AOP) framework. Traditional toxicological studies often rely on phenomenological observations, struggling to connect molecular binding events to ultimate disease states [21]. The AOP-informed, hypothesis-driven model constructed herein aims to bridge this gap by mapping a logical sequence of biologically plausible events.

The framework initiates with the Molecular Initiating Events (MIEs)—the predicted binding-site overlap of DBP and Hesperetin to the PPARG receptor and TNF cytokine. This is hypothesized to trigger the subsequent Cellular Key Events (KEs), namely the perturbation of the Lipid and atherosclerosis pathway, the dysregulation of lipid metabolism, and the activation of the TP53/CASP3 apoptosis axis. Finally, these cellular events are theorized to manifest as the Tissue-level Adverse Outcome (AO) of hepatic steatosis and MASLD. By mapping these putative Key Event Relationships (KERs), this AOP not only outlines the theoretical pathogenesis of DBP but also highlights the potential structural intervention nodes of Hesperetin. This hypothesis-driven model significantly enhances the interpretability of in silico data, offering a modern, systems-biology approach to identifying therapeutic targets for EDC-associated diseases [40].

### 4.6. Limitations and Future Directions

While this integrated computational strategy provides a comprehensive theoretical framework and proposes potential therapeutic mechanisms, the study is inherently limited by its in silico nature. Major limitations include the absolute absence of experimental validation and the reliance on computational predictions rather than physical biochemical binding assays. Although we have computationally evaluated the primary active metabolites, their dynamic in vivo pharmacokinetics cannot be fully captured in silico. The complex pharmacokinetics, tissue distribution, and biotransformation of both DBP and hesperetin cannot be fully captured through static topological networks and molecular docking alone. Additionally, the molecular docking in this study adopts a rigid receptor model, which cannot fully capture the conformational flexibility of proteins and the induced-fit effect upon ligand binding.

To transition this AOP-informed framework from a theoretical paradigm to an empirically supported biological mechanism, comprehensive in vitro and in vivo empirical validations are absolutely imperative. Future research should prioritize validating the functional direction of this regulation through biochemical binding assays, molecular dynamics (MD) simulations, and metabolite-aware physiological models. Explicitly evaluating the dynamic binding and pathway-level modulation of both the parent compounds and their active metabolites will be essential to ascertain whether these in silico hypotheses translate into viable biological interventions against plasticizer-induced metabolic disorders.

## 5. Conclusions

In conclusion, this study integrated network toxicology, network pharmacology, molecular docking, and an AOP-informed, hypothesis-driven framework to systematically explore the potential modulatory mechanisms of Hesperetin against DBP-induced MASLD. Our findings identify PPARG, TNF, and related metabolic–inflammatory pathways (specifically the “Lipid and atherosclerosis” pathway) as computationally prioritized candidate targets. Crucially, while molecular docking simulations suggest structural compatibility and a thermodynamic potential for receptor occupancy at these shared nodes, it must be explicitly noted that these computational findings do not demonstrate direct biological binding, physiological competitive antagonism, or functional hepatoprotective activity.

Therefore, this dual-network paradigm and the resulting AOP-informed, hypothesis-driven model serve strictly as a hypothesis-generating framework. The structural predictions generated herein require comprehensive in vitro and in vivo experimental validation—such as utilizing advanced 3D human liver organoids and high-resolution lipidomics—to ascertain whether these in silico hypotheses translate into viable biological interventions for mitigating EDC-associated metabolic health risks.

## Figures and Tables

**Table 1:** Pathways corresponding to different codes.

Code	Pathway
P1	Lipid and atherosclerosis
P2	Pathways in cancer
P3	Chemical carcinogenesis-receptor activation
P4	PPAR signaling pathway
P5	Prostate cancer
P6	Diabetic cardiomyopathy
P7	Malaria
P8	Efferocytosis
P9	NF-kappa B signaling pathway
P10	Serotonergic synapse

**Table 2:** Information on the PPI targets induced by DBP in MASLD of the top 20.

	Target	Closeness Centrality	Degree	Betweenness Centrality	Topological Coefficient
1	IL6	0.533333333	45	0.08065948	0.200634921
2	TP53	0.52892562	45	0.10011327	0.18403452
3	CTNNB1	0.535564854	42	0.102905707	0.186433064
4	IL1B	0.52892562	42	0.055078959	0.208445642
5	TNF	0.524590164	42	0.05964131	0.209020147
6	AKT1	0.520325203	39	0.061251819	0.208514756
7	MMP9	0.496124031	37	0.043411789	0.217308217
8	PPARG	0.516129032	35	0.111002248	0.186381842
9	EGFR	0.494208494	33	0.039341264	0.232121212
10	CASP3	0.457142857	30	0.014393911	0.268992248
11	BCL2	0.460431655	29	0.029354592	0.264106583
12	CXCL8	0.447552448	29	0.01714914	0.284693019
13	ESR1	0.5	28	0.078522697	0.201819407
14	PTGS2	0.509960159	26	0.069053006	0.252293578
15	CCL2	0.453900709	25	0.006965572	0.270909091
16	IFNG	0.442906574	25	0.004179455	0.323373494
17	PPARA	0.486692015	25	0.1416588	0.15257732
18	ICAM1	0.455516014	23	0.005496859	0.283803153
19	MAPK3	0.450704225	23	0.005629647	0.300889328
20	RELA	0.475836431	23	0.009996874	0.267391304

**Table 3:** Pathways corresponding to different codes.

Number	Pathway
P1	Pathways in cancer
P2	Endocrine resistance
P3	Human papillomavirus infection
P4	MicroRNAs in cancer
P5	Type II diabetes mellitus
P6	Thermogenesis
P7	African trypanosomiasis

**Table 4:** Information on the PPI target for Hesperetin treatment of MASLD of the top 20.

	Target	Closeness Centrality	Degree	Betweenness Centrality	Topological Coefficient
1	ESR1	0.565217391	16	0.264472236	0.222916667
2	HSP90AA1	0.557142857	15	0.162706739	0.227956989
3	TNF	0.513157895	13	0.213602743	0.21474359
4	HSP90AB1	0.513157895	12	0.052502765	0.283333333
5	BCL2	0.541666667	11	0.067430627	0.296791444
6	MDM2	0.475609756	9	0.050609227	0.341880342
7	PPARG	0.506493506	9	0.08182442	0.285185185
8	GSK3B	0.458823529	8	0.074059931	0.347826087
9	MTOR	0.4875	8	0.022632769	0.349137931
10	PTGS2	0.448275862	8	0.047011663	0.346590909
11	MMP9	0.493670886	8	0.068966955	0.297413793
12	MMP2	0.469879518	7	0.02068215	0.367346939
13	RELA	0.493670886	7	0.024934547	0.322580645
14	AR	0.443181818	6	0.022756775	0.403846154
15	PIK3CA	0.458823529	6	0.050671551	0.314102564
16	AHR	0.438202247	5	0.03325258	0.408
17	FGFR1	0.40625	5	0.033558255	0.282352941
18	SERPINE1	0.410526316	5	5.40E-04	0.45
19	CYP1A2	0.351351351	4	0.010931783	0.40625
20	CYP1A1	0.40625	4	0.02230629	0.35

**Table 5:** Redocking validation of the selected protein structures using their co-crystallized native ligands.

Target Protein	PDB ID	Co-Crystallized Native Ligand (PubChem ID)	RMSD (Å)
PPARG	3E00	10308	0.34126
TNF	2AZ5	16078	0.28741
AKT1	6HHF	135413508	0.39652

Note: RMSD represents the root-mean-square deviation of the co-crystallized native ligand after redocking. TP53, CASP3, and HSP90AA1 are not listed due to the absence of co-crystallized native small-molecule ligands in their corresponding PDB structures.

**Table 6:** Predicted docking scores of DBP and hesperetin with the selected protein targets.

Protein (PDB ID)	Ligand	Binding Pocket	Affinity (Mean ± SD, kcal mol^−1^)
PPARG (3E00)	DBP	C1	−6.90 ± 0.00
	Hesperetin	C1	−8.20 ± 0.00
TNF (2AZ5)	DBP	C3	−6.27 ± 0.31
	Hesperetin	C3	−7.4
AKT1 (6HHF)	DBP	C1	−7.4
CASP3 (1NMQ)	DBP	C1	−6.2
TP53 (1TUP)	DBP	C2	−5.5
HSP90AA1 (5FWL)	Hesperetin	C3	−8.8

Note: Affinities for the core shared targets (PPARG and TNF) are reported as Mean ± SD based on independent triplicate runs. Affinities for other single-network targets (AKT1, CASP3, TP53, HSP90AA1) represent the top-pose score from a single optimized docking run.

## Data Availability

The original contributions presented in this study are included in the article/Supplementary Material. Further inquiries can be directed to the corresponding authors.

## Supplementary Materials

The following supporting information can be downloaded at: https://www.mdpi.com/article/10.3390/nu18152577/s1, Figure S1: Rank heatmap of 129 intersecting targets across eight topological algorithms of DBP-MASLD; Figure S2: Rank heatmap of 44 intersecting targets across eight topological algorithms of Hesperetin-MASLD; Figure S3. (A) Mono-n-butyl phthalate (MBP) docked with TNF (Pocket C3, −6.30 ± 0.00 kcal/mol). (B) MBP docked with PPARG (Pocket C1, −6.33 ± 0.06 kcal/mol). (C) Hesperetin-7-O-glucuronide docked with TNF (Pocket C3, −8.30 ± 0.00 kcal/mol). (D) Hesperetin-7-O-glucuronide docked with PPARG (Pocket C1, −7.20 ± 0.00 kcal/mol). The robust triplicate data confirmed successful embedding into the shared active pockets without severe steric hindrance; Figure S4. Receiver Operating Characteristic (ROC) curves validating the AutoDock Vina docking protocol using active and matched decoy datasets from the DUD-E database. (A) Enrichment performance for PPARG (AUC = 0.614). (B) Enrichment performance for TNF (AUC = 0.834). Table S1: Information on the PPI target induced by DBP in MASLD; Table S2: Information on the PPI target for Hesperetin treatment of MASLD; Table S3: Per-Algorithm Rankings (Top 15) for DBP-MASLD Intersecting Targets; Table S4: Per-Algorithm Rankings (Top 15) for Hesperetin-MASLD Intersecting Targets; Table S5: Complete Gene Ontology (GO) enrichment results of DBP-MASLD intersecting targets; Table S6: Complete KEGG pathway enrichment results of DBP-MASLD intersecting targets; Table S7: Complete Gene Ontology (GO) enrichment results of Hesperetin-MASLD intersecting targets; Table S8: Complete KEGG pathway enrichment results of Hesperetin-MASLD intersecting targets; Table S9: Per-Algorithm Rankings (Top 15) for DBP-MASLD targets under stringent sensitivity thresholds; Table S10: Per-Algorithm Rankings (Top 15) for Hesperetin-MASLD targets under stringent sensitivity thresholds; Sensitivity Analysis. (Note: Tables S5–S10 are provided as a single separate Excel file).
