# Supplementary material for: Computational Prediction of Hesperetin Modulatory Targets in Dibutyl Phthalate-Associated Steatotic Liver Injury: An Integrated Network Toxicology, Molecular Docking, and AOP-Based Study
Source: Nutrients. 2026 Aug 6;18(15):2577. doi: 10.3390/nu18152577 (PMC13468542; doi:10.3390/nu18152577)
Supplement: Supplementary file 1 [file nutrients-18-02577-s001.zip › Supplementary Figures.pdf]

Supplementary Materials

Computational Prediction of Hesperetin Modulatory Targets in Dibutyl Phthalate-Associated Steatotic Liver Injury: An Integrated Network Toxicology, Molecular Docking, and AOP-Based Study

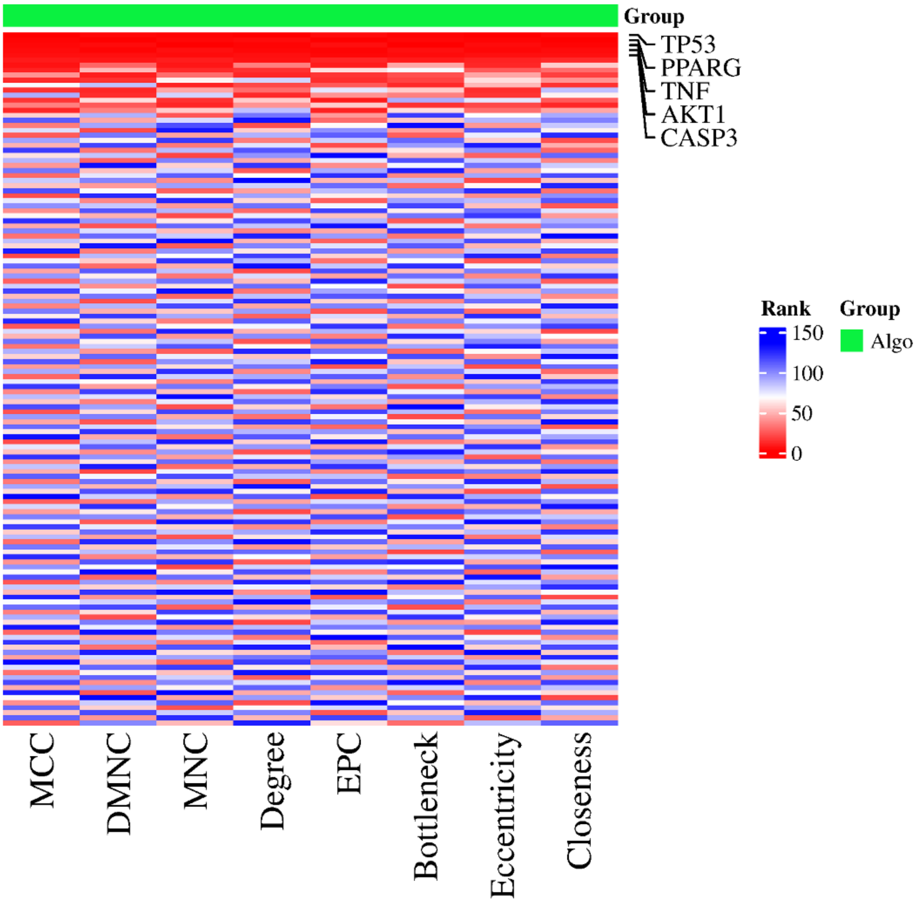

Figure S1. Rank heatmap of 129 intersecting targets across eight topological algorithms of DBP-MASLD. The heatmap visualizes the specific ranking of each target within the PPI network evaluated by MCC, DMNC, MNC, Degree, EPC, Bottleneck, Eccentricity, and Closeness algorithms. The color gradient from red to blue represents the rank value, where deep red indicates a top-ranking position (closer to rank 1) and deep blue indicates a lower position. The five selected core targets (TP53, PPARG, TNF, AKT1, and CASP3) consistently rank at the top across all eight topological dimensions (forming the solid red band at the top), structurally supporting their selection as candidate critical hub genes and helping to mitigate subjective screening bias.

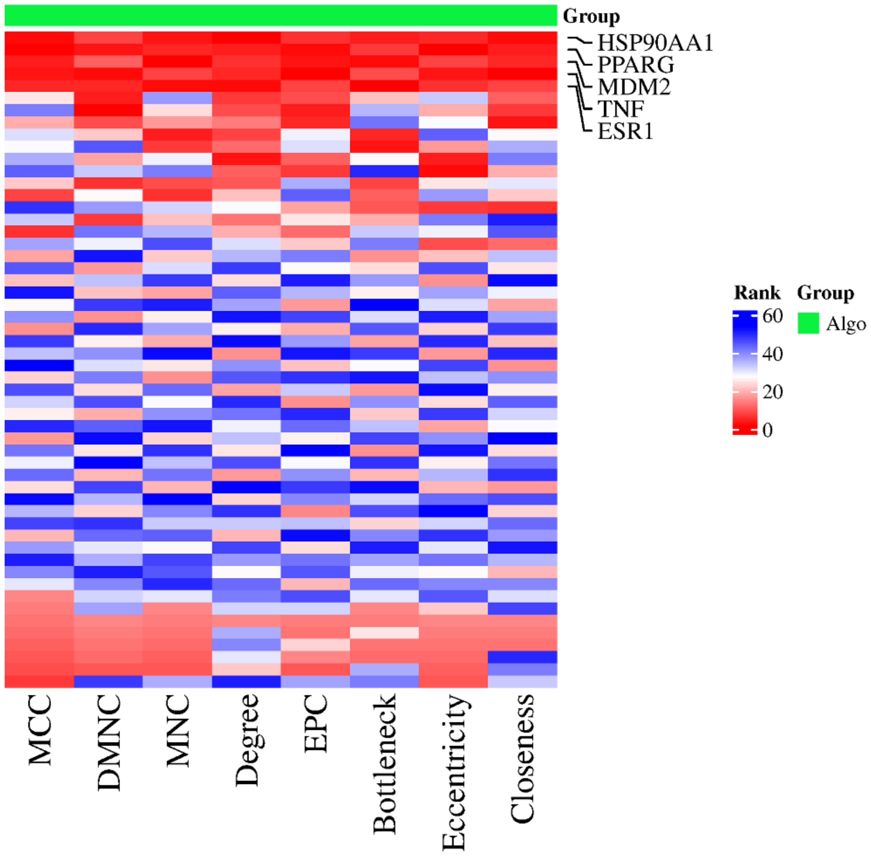

Figure S2. Rank heatmap of 40 intersecting targets across eight topological algorithms of Hesperetin-MASLD. The heatmap visualizes the specific ranking of each target within the PPI network evaluated by MCC, DMNC, MNC, Degree, EPC, Bottleneck, Eccentricity, and Closeness algorithms. The color gradient from red to blue represents the rank value, where deep red indicates a top-ranking position (closer to rank 1) and deep blue indicates a lower position. The five selected core targets (HSP90AA1, PPARG, MDM2, TNF, and ESR1) consistently rank at the top across all eight topological dimensions (forming the solid red band at the top), structurally supporting their selection as candidate critical hub genes and helping to mitigate subjective screening bias.

Table S1. Information on the PPI target induced by DBP in MASLD

	Target	Closeness Centrality	Degree	Betweenness Centrality	Topological Coefficient
1	IL6	0.533333333	45	0.08065948	0.200634921
2	TP53	0.52892562	45	0.10011327	0.18403452
3	CTNNB1	0.535564854	42	0.102905707	0.186433064
4	IL1B	0.52892562	42	0.055078959	0.208445642
5	TNF	0.524590164	42	0.05964131	0.209020147

6	AKT1	0.520325203	39	0.061251819	0.208514756
7	MMP9	0.496124031	37	0.043411789	0.217308217
8	PPARG	0.516129032	35	0.111002248	0.186381842
9	EGFR	0.494208494	33	0.039341264	0.232121212
10	CASP3	0.457142857	30	0.014393911	0.268992248
11	BCL2	0.460431655	29	0.029354592	0.264106583
12	CXCL8	0.447552448	29	0.01714914	0.284693019
13	ESR1	0.5	28	0.078522697	0.201819407
14	PTGS2	0.509960159	26	0.069053006	0.252293578
15	CCL2	0.453900709	25	0.006965572	0.270909091
16	IFNG	0.442906574	25	0.004179455	0.323373494
17	PPARA	0.486692015	25	0.1416588	0.15257732
18	ICAM1	0.455516014	23	0.005496859	0.283803153
19	MAPK3	0.450704225	23	0.005629647	0.300889328
20	RELA	0.475836431	23	0.009996874	0.267391304
21	TGFB1	0.457142857	22	0.004537814	0.305962854
22	HSP90AB1	0.463768116	21	0.014405691	0.243986254
23	MAPK1	0.465454545	20	0.00363787	0.278061224
24	SERPINE1	0.426666667	20	0.011124226	0.294078947
25	SREBF1	0.447552448	20	0.038092434	0.180952381
26	CD36	0.432432432	19	0.017657023	0.227631579
27	MAPK14	0.438356164	19	0.004527184	0.333126935
28	MAPK8	0.442906574	19	0.005864406	0.322444041
29	MMP2	0.429530201	19	0.00561998	0.330921053
30	CXCR4	0.42384106	18	0.005127932	0.315752461
31	SCARB1	0.428093645	18	0.027136398	0.233618234
32	TNFRSF1A	0.411575563	17	0.003583947	0.349121467
33	VCAM1	0.418300654	17	0.002506535	0.349717969
34	PTPRC	0.429530201	16	0.003383483	0.322408537

35	APP	0.422442244	15	0.033402181	0.309333333
36	MMP3	0.384384384	15	0.002985383	0.386021505
37	CYP3A4	0.331606218	14	0.014400954	0.303571429
38	FABP4	0.401253918	14	0.008459818	0.233333333
39	PARP1	0.426666667	14	0.002480524	0.316202091
40	CYP1A1	0.414239482	13	0.047844772	0.174825175
41	GSK3B	0.429530201	13	0.006877118	0.275403609
42	LIPE	0.391437309	13	0.036719007	0.2191582
43	RXRA	0.439862543	13	0.03446988	0.194570136
44	SP1	0.428093645	13	0.003674579	0.279549719
45	CDK4	0.415584416	12	0.005037819	0.288812785
46	CYP2C9	0.379821958	12	0.036080629	0.217391304
47	FASN	0.403785489	12	0.005372675	0.248737374
48	NR1I2	0.346883469	11	0.031013605	0.269794721
49	UGT1A1	0.329048843	11	0.011034335	0.348484848
50	PLAU	0.373177843	10	5.47E-04	0.455172414
51	AR	0.4	9	0.015824782	0.388059701
52	CXCR2	0.372093023	9	2.06E-04	0.51532567
53	CYP1A2	0.326530612	9	0.001461154	0.393518519
54	MAPK9	0.379821958	9	0.001263397	0.378136201
55	NOS3	0.405063291	9	0.003033724	0.334834835
56	PRKCA	0.408945687	9	0.002745224	0.353276353
57	CDK1	0.382089552	8	6.95E-04	0.4140625
58	CYP2C19	0.373177843	8	0.01189151	0.266666667
59	CYP2D6	0.374269006	8	0.014280101	0.261111111
60	FLT1	0.379821958	8	8.58E-04	0.390873016
61	LPL	0.408945687	8	0.001317017	0.315
62	MMP7	0.405063291	8	6.70E-04	0.388513514
63	NLRP3	0.391437309	8	0.001326988	0.429435484

64	NR1H3	0.377581121	8	0.001152019	0.322916667
65	TLR9	0.367816092	8	1.27E-04	0.522727273
66	TNFSF11	0.385542169	8	2.53E-04	0.474637681
67	ABCA1	0.380952381	7	4.15E-04	0.379120879
68	ACE	0.367816092	7	9.19E-04	0.418367347
69	ALDH1A1	0.393846154	7	0.039815167	0.249394673
70	CASP7	0.361581921	7	3.72E-04	0.391534392
71	CTSB	0.36056338	7	0.001100697	0.334231806
72	CTSS	0.372093023	7	9.39E-04	0.363196126
73	F2	0.367816092	7	0.011362329	0.357142857
74	MMP13	0.366762178	7	8.08E-04	0.515873016
75	VIM	0.395061728	7	8.16E-04	0.428571429
76	ABCG1	0.369942197	6	3.35E-04	0.40070922
77	AGTR1	0.346883469	6	0.001249325	0.333333333
78	BIRC5	0.354570637	6	1.89E-04	0.382978723
79	DNMT1	0.383233533	6	8.85E-05	0.466101695
80	FOXM1	0.378698225	6	6.71E-04	0.391666667
81	MIF	0.385542169	6	0.001268223	0.355392157
82	PCK1	0.35359116	6	0.001391112	0.328947368
83	PLIN1	0.363636364	6	1.47E-04	0.402173913
84	PPARD	0.376470588	6	3.25E-04	0.413333333
85	ABCB1	0.298368298	5	5.64E-05	0.53
86	ADAM17	0.361581921	5	3.40E-04	0.392156863
87	COL18A1	0.338624339	5	1.49E-04	0.435897436
88	GPAM	0.368876081	5	3.85E-04	0.426086957
89	HNF4A	0.412903226	5	0.013194792	0.346666667
90	NR1I3	0.330749354	5	0.001212815	0.420689655
91	PLIN2	0.375366569	5	7.97E-05	0.439215686
92	PRKCE	0.340425532	5	6.13E-04	0.378947368

93	AHR	0.367816092	4	0.002972654	0.394444444
94	CES1	0.306220096	4	0.007298082	0.384615385
95	CNR1	0.256513026	4	0.004109404	0.55
96	ELANE	0.343163539	4	4.05E-04	0.448863636
97	FABP1	0.342245989	4	1.45E-05	0.558823529
98	GPR55	0.335958005	4	0.047763309	0.303571429
99	PTPN1	0.382089552	4	1.23E-05	0.573770492
100	SIRT2	0.379821958	4	0	0.603448276
101	VCP	0.368876081	4	4.25E-05	0.509090909
102	ABCC2	0.263374486	3	0	0.705882353
103	CES2	0.258064516	3	0	0.644444444
104	CNR2	0.253968254	3	0	0.733333333
105	EPHX2	0.294252874	3	0.031003937	0.476190476
106	FAAH	0.253968254	3	0	0.733333333
107	HSD11B1	0.298368298	3	0	0.648148148
108	KRT7	0.35359116	3	0.005258073	0.353741497
109	PPP1CA	0.355555556	3	4.16E-04	0.424242424
110	TRIB3	0.398753894	3	2.55E-04	0.512077295
111	ADRB3	0.335078534	2	0	0.655172414
112	AFP	0.285077951	2	2.14E-04	0.5
113	ALDH2	0.228571429	2	0.015625	0.5
114	GRM5	0.304038005	2	0.012768187	0.5
115	HTR2A	0.245210728	2	0.001393121	0.5
116	PCK2	0.332467532	2	2.21E-04	0.58
117	PLIN5	0.335078534	2	0	0.655172414
118	STAR	0.31372549	2	0.001887619	0.5
119	TSPO	0.324873096	2	0.001620899	0.5
120	ABCC3	0.258064516	1	0	0
121	AKR1B1	0.186317322	1	0	0

122	FFAR1	0.341333333	1	0	0
123	GDF15	0.346883469	1	0	0
124	PRKAA2	0.281938326	1	0	0
125	PTPN2	0.331606218	1	0	0
126	PTX3	0.348773842	1	0	0
127	SHBG	0.334203655	1	0	0
128	SOAT1	0.300469484	1	0	0
129	TGFB1I1	0.286353468	1	0	0

Table S2. Information on the PPI target for Hesperetin treatment of MASLD.

	Target	Closeness Centrality	Degree	Betweenness Centrality	Topological Coefficient
1	ESR1	0.565217391	16	0.264472236	0.222916667
2	HSP90AA1	0.557142857	15	0.162706739	0.227956989
3	TNF	0.513157895	13	0.213602743	0.21474359
4	HSP90AB1	0.513157895	12	0.052502765	0.283333333
5	BCL2	0.541666667	11	0.067430627	0.296791444
6	MDM2	0.475609756	9	0.050609227	0.341880342
7	PPARG	0.506493506	9	0.08182442	0.285185185
8	GSK3B	0.458823529	8	0.074059931	0.347826087
9	MTOR	0.4875	8	0.022632769	0.349137931
10	PTGS2	0.448275862	8	0.047011663	0.346590909
11	MMP9	0.493670886	8	0.068966955	0.297413793
12	MMP2	0.469879518	7	0.02068215	0.367346939
13	RELA	0.493670886	7	0.024934547	0.322580645
14	AR	0.443181818	6	0.022756775	0.403846154
15	PIK3CA	0.458823529	6	0.050671551	0.314102564
16	AHR	0.438202247	5	0.03325258	0.408
17	FGFR1	0.40625	5	0.033558255	0.282352941
18	SERPINE1	0.410526316	5	5.40E-04	0.45

19	CYP1A2	0.351351351	4	0.010931783	0.40625
20	CYP1A1	0.40625	4	0.02230629	0.35
21	APP	0.414893617	4	0.046296296	0.3
22	RIPK1	0.443181818	4	0.005438817	0.451923077
23	TERT	0.386138614	4	3.94E-04	0.611111111
24	SNCA	0.386138614	3	0.024212337	0.392156863
25	KDM1A	0.386138614	3	0.051282051	0.431372549
26	IDO1	0.39	3	0.020715991	0.392156863
27	PTK2	0.419354839	3	0	0.536231884
28	TYK2	0.40625	3	0.055862948	0.333333333
29	TTR	0.319672131	2	0	0.7
30	XDH	0.330508475	2	0.004498426	0.5
31	RPS6KA3	0.382352941	2	0.003638905	0.558823529
32	PKM	0.348214286	2	0.001180837	0.545454545
33	TLR9	0.382352941	2	0.008934194	0.5
34	DNMT1	0.28057554	1	0	0
35	ELANE	0.333333333	1	0	0
36	SHBG	0.364485981	1	0	0
37	HNF4A	0.317073171	1	0	0
38	MMP13	0.342105263	1	0	0
39	PLIN1	0.339130435	1	0	0
40	PTPN1	0.291044776	1	0	0

Table S3. Per-Algorithm Rankings (Top 15) for DBP-MASLD Intersecting Targets.

Rank	MCC	DMNC	MNC	Degree	EPC	Bottleneck	Eccentricity	Closeness
1	TP53	CASP3	TP53	IL6	TP53	PPARA	CASP3	CTNNB1
2	AKT1	PPARG	TNF	TP53	AKT1	PPARG	PPARG	IL6
3	TNF	TP53	AKT1	CTNNB1	TNF	CTNNB1	TP53	TP53
4	CASP3	TNF	CASP3	IL1B	CASP3	TP53	TNF	IL1B

5	PPARG	AKT1	PPARG	TNF	PPARG	IL6	AKT1	TNF
6	EGFR	SIRT2	IL6	AKT1	LIPE	ESR1	SIRT2	AKT1
7	MMP9	IFNG	CTNNB1	MMP9	RXRA	PTGS2	IFNG	PPARG
8	BCL2	PPARA	IL1B	PPARG	SP1	AKT1	PPARA	PTGS2
9	CXCL8	CCL2	EGFR	EGFR	CDK4	TNF	CCL2	ESR1
10	ESR1	LIPE	MMP9	CASP3	CYP2C9	IL1B	NR1I2	MMP9
11	PTGS2	RXRA	BCL2	BCL2	FASN	LIPE	UGT1A1	EGFR
12	CCL2	SP1	ESR1	CXCL8	NR1I2	CASP3	PLAU	PPARA
13	PPARA	CDK4	PTGS2	ESR1	UGT1A1	BCL2	AR	BCL2
14	IFNG	CYP2C9	CXCL8	PTGS2	PLAU	CXCL8	CXCR2	CASP3
15	SIRT2	FASN	CCL2	CCL2	AR	LPL	CYP1A2	CXCL8

Table S4. Per-Algorithm Rankings (Top 15) for Hesperetin-MASLD Intersecting Targets.

Rank	MCC	DMNC	MNC	Degree	EPC	Bottleneck	Eccentricity	Closeness
1	ESR1	TNF	HSP90AA1	ESR1	MDM2	PPARG	ESR1	ESR1
2	HSP90AA1	MDM2	ESR1	HSP90AA1	PPARG	TNF	MDM2	HSP90AA1
3	TNF	ESR1	PPARG	TNF	ESR1	MDM2	HSP90AA1	BCL2
4	MDM2	PPARG	TNF	HSP90AB1	HSP90AA1	ESR1	PPARG	TNF
5	PPARG	HSP90AA1	MDM2	BCL2	TNF	HSP90AA1	TNF	HSP90AB1
6	AHR	MMP9	AHR	MDM2	IDO1	IDO1	HSP90AB1	PPARG
7	FGFR1	MMP2	FGFR1	PPARG	PTK2	PTK2	BCL2	MMP9
8	SERPINE1	RELA	SERPINE1	GSK3B	TYK2	TYK2	GSK3B	RELA
9	CYP1A2	AR	CYP1A2	MTOR	TTR	TTR	MTOR	MTOR
10	CYP1A1	PIK3CA	CYP1A1	PTGS2	XDH	XDH	PTGS2	MDM2
11	APP	APP	APP	MMP9	RPS6KA3	RPS6KA3	AHR	MMP2
12	RIPK1	RIPK1	RIPK1	MMP2	PKM	PKM	FGFR1	GSK3B
13	TERT	TERT	TERT	RELA	TLR9	TLR9	SERPINE1	PIK3CA
14	SNCA	SNCA	SNCA	AR	DNMT1	DNMT1	CYP1A2	PTGS2
15	KDM1A	KDM1A	KDM1A	PIK3CA	ELANE	ELANE	CYP1A1	AR

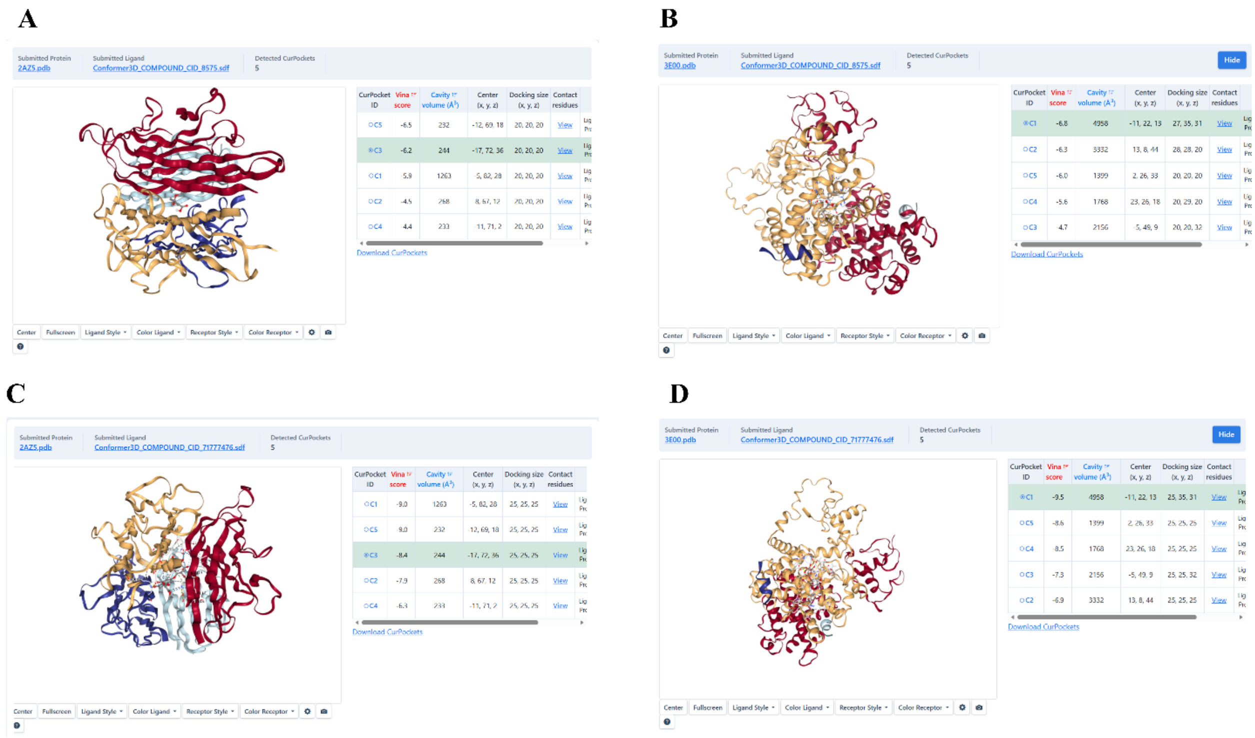

Figure S3. (A) Mono-n-butyl phthalate (MBP) docked with TNF (Pocket C3, -6.30 ± 0.00 kcal/mol). (B) MBP docked with PPARG (Pocket C1, -6.33 ± 0.06 kcal/mol). (C) Hesperetin-7-O-glucuronide docked with TNF (Pocket C3, -8.30 ± 0.00 kcal/mol). (D) Hesperetin-7-O-glucuronide docked with PPARG (Pocket C1, -7.20 ± 0.00 kcal/mol). The robust triplicate data confirmed successful embedding into the shared active pockets without severe steric hindrance.

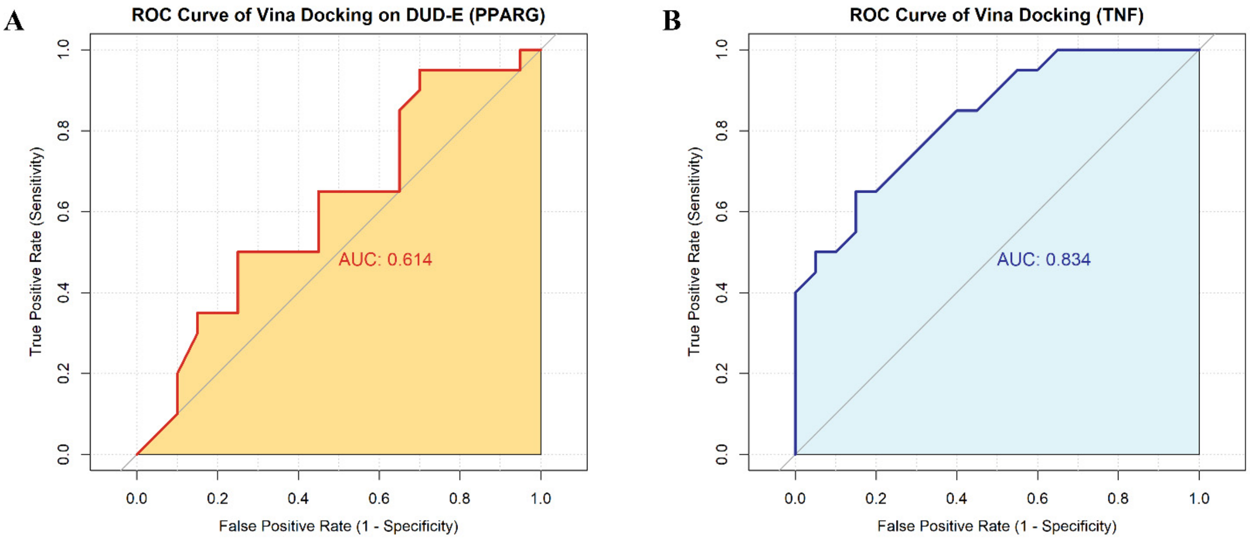

Figure S4. Receiver Operating Characteristic (ROC) curves validating the AutoDock Vina docking protocol using active and matched decoy datasets from the DUD-E database. (A) Enrichment performance for PPARG (AUC = 0.614). (B) Enrichment performance for TNF (AUC = 0.834).
